# Lipid transfer proteins rectify inter-organelle flux and accurately deliver lipids at membrane contact sites

**DOI:** 10.1194/jlr.R085324

**Published:** 2018-06-08

**Authors:** Kentaro Hanada

**Affiliations:** Department of Biochemistry and Cell Biology, National Institute of Infectious Diseases, Shinjuku-ku, Tokyo 162-8640, Japan

**Keywords:** ceramide, cholesterol, disordered region, endoplasmic reticulum, lipid-transfer domain, non-equilibrium thermodynamics, phospholipids, plasma membrane, sphingomyelin

## Abstract

The endoplasmic reticulum (ER) is the main center for the synthesis of various lipid types in cells, and newly synthesized lipids are delivered from the ER to other organelles. In the past decade, various lipid transfer proteins (LTPs) have been recognized as mediators of lipid transport from the ER to other organelles; inter-organelle transport occurs at membrane contact sites (MCSs) and in a nonvesicular manner. Although the intermembrane transfer reaction catalyzed by LTPs is an equilibrium reaction, various types of newly synthesized lipids are transported unidirectionally in cells. This review provides a brief history of the inter-organelle trafficking of lipids and summarizes the structural and biochemical characteristics of the ceramide transport protein (CERT) as a typical LTP acting at MCSs. In addition, this review compares several LTP-mediated inter-organelle lipid trafficking systems and proposes that LTPs generate unidirectional fluxes of specific lipids between different organelles by indirect coupling with the metabolic reactions that occur in specific organelles. Moreover, the available data also suggest that the major advantage of LTP-mediated lipid transport at MCSs may be the accuracy of delivery. Finally, how cholesterol is enriched in the plasma membrane is discussed from a thermodynamic perspective.

Eukaryotic cells have various membrane-bound distinct compartments, called organelles, with specific functions. Subcellular compartmentalization appears to have been a fundamental event in the evolution of life, enabling the manipulation of numerous metabolic reactions as well as macromolecule functions in the cell. The functions of each organelle may be largely attributable to specific sets of proteins localized there. However, lipids are also the major constituents of all cell membranes and play crucial roles in organelle structure and function (1–3). Highly diverse molecular species of lipids exist in biological organisms (see global databases: LipidBank, http://lipidbank.jp/ and LIPID MAPS, http://lipidmaps.org/resources/tutorials/databases.html). The chemical backbones of major lipid classes in biomembranes are classified into three types (**Fig. 1A**): acylated glycerols (glycerolipids), acylated long-chain bases (sphingolipids), and sterols, and each type consists of various subtypes, for example, glycerolipids include neutral glycerolipids [e.g., diacylglycerol (DAG)], glycosylglycerolipids (e.g., galactosyldiacylglycerol), and glycerophospholipids.

**Fig. 1.**
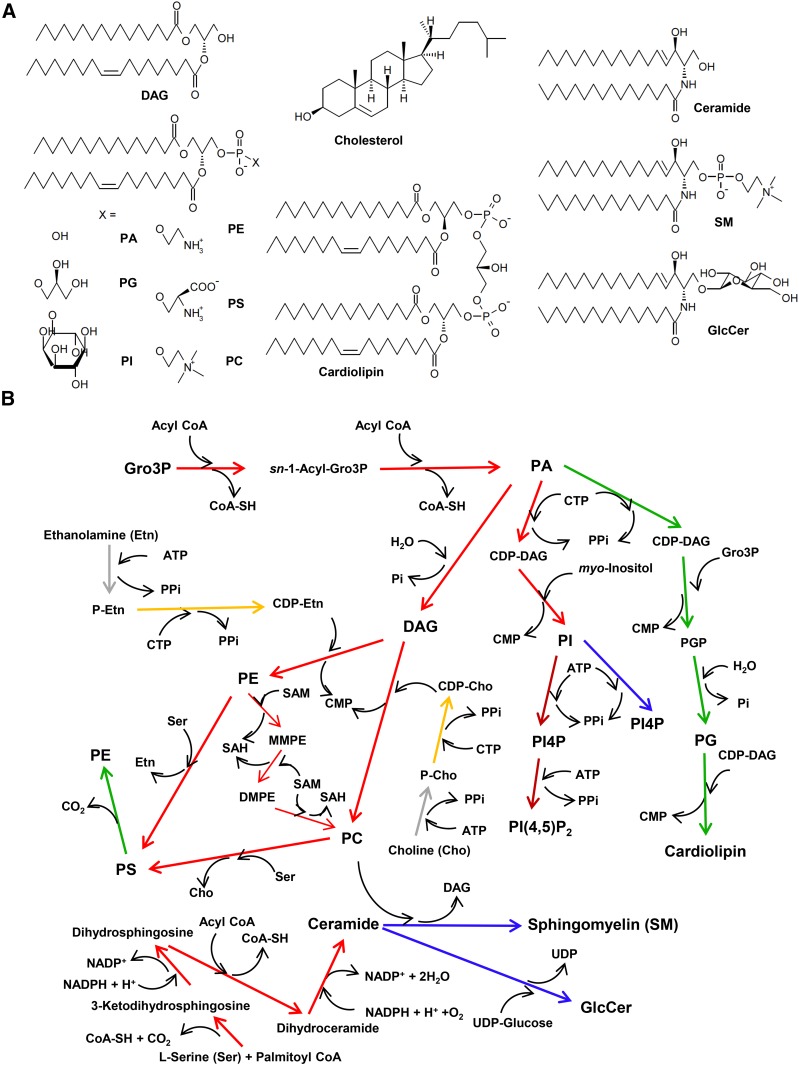
Biosynthesis of glycerophospholipids and sphingolipids in mammalian cells. A: The structures of major lipid types in mammalian cells are shown. For simplicity, the diradyl moieties of glycerolipids and sphingolipids are depicted as *sn*-1-palmitoyl-2-oleoyl (C16:0/C18:1) and *N*-palmitoyl d18:1-sphingosine (C16-ceramide), respectively, as their typical structures, although variations in diradyl structures occur in cells. B: The biosynthetic pathway of major glycerophospholipids and sphingolipids in typical mammalian cells is shown. Arrow colors represent the organelles in which the reactions predominantly occur: red, ER; orange, nuclear envelope; blue, Golgi apparatus; green, mitochondria; brown, PM; gray, cytosol. Conversion of DAG to PC may also occur in the Golgi apparatus (see text and Fig. 5A). The methylation of PE to produce PC is specific to hepatocytes. Whereas de novo synthesis of PI4P occurs in the Golgi apparatus and PM by different PI-4 kinase isoforms, PI4P of the PM is often converted to PI(4,5)P_2_ by a PM-localizing PI4P-5 kinase. Gro3P, glycerol-3-phosphate; Pi, inorganic phosphate; PPi, pyrophosphate; P-Cho, phosphocholine; P-Etn, phosphoethanolamine; PGP, PG-1-phosphate; MMPE, *N*-monomethyl PE; DMPE, *N*-dimethyl PE; SAM, *S*-adenosyl- l-methionine; SAH, *S*-adenosyl- l-homocysteine.

Because the endoplasmic reticulum (ER) is the main center for the synthesis of diverse lipid types (4, 5), the cell must have a system to transport newly synthesized lipids from the ER to other organelles (Fig. 1B) (6). Moreover, after being transported from the ER to specific organelles, several lipid types are metabolically converted to other lipid types (Fig. 1B), which is required to produce a complete lipidome of cells. Therefore, inter-organelle trafficking of lipids is assumed to be generally essential for the life of eukaryotes. In the past decade, various types of lipid transfer proteins (LTPs) have been recognized to mediate the transport of a number of lipid types from the ER to other organelles at membrane contact sites (MCSs) in nonvesicular manners (**Table 1**) (2, 7, 8).

**TABLE 1.** LTPs that mediate the inter- or intra-organelle transport of lipids at MCSs in mammalian cells

Human LTP (Yeast Ortholog)	LTD Family	Lipid*^a^*	MCS	References
CERT	START	Ceramide/DAG	ER-Golgi	(46, 65, 66)
OSBP	ORD	Sterol/PI4P	ER-Golgi	(99)
Nir2	PITD	PI/PC	ER-Golgi	(81, 106)
		PI/PA	ER-PM	(104, 107, 108)
FAPP2	GLTD	GlcCer	Intra-Golgi (or Golgi-TGN)	(133, 134)
			ER-Golgi	(45)
C1P-transfer protein	GLTD	C1P	TGN-PM (?)	(135)
ORP5 and -8 (Osh6 and -7)	ORD	PS/PI4P	ER-PM	(100, 101, 109, 111)
GRAMD1 isoforms (Lam/Ysp/Ltc isoforms)	VASt	Sterol	ER-PM, ER-Mito and/or ER-vacuole	(136, 137, 141–145)
STARD7	START	PC	EM-Mito	(123)
StAR	START	Sterol	OM-IM of Mito	(40)
PRELID1 (Ups1) + TRIAP1 (Mdm35)	*^b^*	PA	OM-IM of Mito	(120–122, 124–126)
PRELID2 (Ups2) + TRIAP1 (Mdm35)	*^b^*	PS	OM-IM of Mito	(125, 127, 128)
E-Syt2	SMP	Various glycerophospholipids	ER-PM	(149, 151)
PDZD8 (?) (Mmm1+Mdm12+Mdm34)*^c^*	SMP	Various glycerophospholipids	ER-Mito (in yeast)	(153–156, 159)
TEX2/HT008 (?) (Nvj2)	SMP	Sterol (?)	NE-vacuole (in yeast)	(160)
		Ceramide (?)	ER-Golgi (in yeast)	(161)
STARD3/MLN64	START	Sterol	ER-LE/LY	(180)
ORP1L	ORD	Sterol	ER-LE/LY	(182–184)
α-Tocopherol-transfer protein	CRAL-TRIO/Sec14	α-Tocopherol (vitamin E)	LE/LY-PM (?)	(185, 226)

CRAL-TRIO, cellular retinaldehyde-binding protein and TRIO guanine exchange factor; PITD, PI-transfer domain; GLTD, glycolipid-transfer domain; TGN, *trans* Golgi network; OM-IM of Mito, from the outer membrane to the inner membrane of mitochondria; NE, nuclear envelope; MLN64, metastatic lymph node 64. “(?)” represents “currently speculative.”

a The slash represents “co-exchanging” lipid type. Note that GRAMD1b might mediate sterol/PI(4,5)P_2_ coexchange (145).

b LTD composed of two different subunits.

c Mmm1, Mdm12, Mdm34, and non-SMP protein Mdm10 forms the ERMES complex in yeast.

LTPs are defined as proteins capable of transferring lipids between different membranes. The inter-membrane lipid-transfer reaction catalyzed by lipid-transfer domains (LTDs) is essentially an equilibrium reaction: when an LTP is added to a system with multiple membranes with different concentrations of a lipid, the LTP catalyzes the inter-membrane transfer of its ligand lipid toward the equilibrium state, in which the concentrations of the lipid in different membranes are equal. Nevertheless, in living cells, various types of newly synthesized lipids appear to be transported unidirectionally. Thus, to achieve the thermodynamic non-equilibrium transport of lipids, additional factors are required. This review provides a brief history of the inter-organelle trafficking of lipids and summarizes the structural and biochemical characteristics of the ceramide transport protein (CERT) as a typical LTP acting at MCSs. In addition, this review compares several LTP-mediated inter-organelle lipid trafficking systems and proposes that LTPs generate unidirectional fluxes of specific lipids between different organelles by indirect coupling with the metabolic reactions that occur in specific organelles. Moreover, the available data also suggest that the major advantage of LTP-mediated lipid transport at MCSs may be the accuracy of delivery. Finally, how cholesterol is enriched in the plasma membrane (PM) is discussed from a thermodynamic perspective.

## STRUCTURE AND BIOSYNTHETIC PATHWAYS OF MAJOR LIPID TYPES IN MAMMALIAN CELLS

Because a comprehensive description of inter-organelle transport of lipids is beyond the scope of this review, a focus is here placed on inter-organelle translocation of major lipid types in their biosynthesis in mammalian cells. In mammalian cells (as well as other eukaryotic cells), several glycerophospholipid classes are ubiquitous: phosphatidylcholine (PC), phosphatidylethanolamine (PE), phosphatidylserine (PS), phosphatidylinositol (PI), phosphatidylglycerol (PG), and cardiolipin. Except for cardiolipin (which is exclusively localized to mitochondria), the other five classes are widely distributed to most of the organelles, although phospholipid composition deviates depending on the organelle type and the state of cells. PC is the most abundant phospholipid class (∼50% of total phospholipids) in eukaryotes from fungi to humans.

The biosynthetic pathways of the major phospholipids in mammalian cells are depicted in Fig. 1B (4, 5, 9). PC and PE are largely synthesized by the pathway with CDP-alcohol intermediates, which is referred to as the CDP-alcohol or Kennedy pathway (Fig. 1). In this pathway, choline and ethanolamine are phosphorylated, and the resultant phosphocholine and phosphoethanolamine are converted to CDP-choline and CDP-ethanolamine, respectively. These CDP-alcohols are then conjugated with *sn*-1,2-DAG to produce PC and PE (CDP-choline/CDP-ethanolamine + DAG → PC/PE + CMP). The conjugation reaction is catalyzed by choline/ethanolamine phosphotransferase (CEPT) or choline phosphotransferase (CPT). CEPT, which is capable of using both CDP-choline and CDP-ethanolamine, is ubiquitously expressed in human tissues, while the expression of CPT, which is specific to CDP-choline, is relatively tissue-specific (e.g., abundant in the testis) (10, 11). CEPT is likely to be the predominant isoform to produce PC in most cell types, while CPT may play tissue-specific roles. Although CEPT and CPT are structurally similar, they exhibit different organelle distributions (10, 12): CEPT is largely distributed in the ER and nuclear envelope, while CPT is in the Golgi apparatus (12). PC is also synthesized by the methylation of PE in the ER in the liver.

PI and PG are synthesized by the CDP-DAG pathway, which differs from the CDP-alcohol pathway (Fig. 1B). The CDP-DAG intermediate is synthesized by the catalytic reaction [phosphatidic acid (PA) + CTP → CDP-DAG + pyrophosphate] of CDP-DAG synthases. There are three isoforms of CDP-DAG synthases in mammalian cells: two isoforms (CDP-DAG synthase 1 and 2) localize to the ER, and one (mammalian homolog of Tam41) localizes to mitochondria (5, 13). PI is synthesized by PI synthase (CDP-DAG + *myo*-inositol → PI + CMP) in the ER. After being transported to other organelles, PI is phosphorylated to produce various phosphoinositide species, which often serve as important modulators in the formation and function of organelles (14, 15). The synthesis of PG and cardiolipin occurs in the inner membrane of mitochondria: PA is transported from the ER to mitochondria (as described in the Other LTP-Mediated Pathways that Act at MCSs section) and is converted to CDP-DAG by Tam41 (13). Then, CDP-DAG is converted to PG-1-phosphate (CDP-DAG + glycerol-3-phosphate → PG-1-phosphate + CMP) by its synthase. PG-1-phosphate is dephosphorylated by PG-1-phosphate phosphatase to produce PG, which is converted to cardiolipin (PG + CDG-DAG → cardiolipin + CMP) by cardiolipin synthase. PG is moved from the inner to the outer mitochondrial membrane, and then to the ER, while cardiolipin is restricted to the inner mitochondrial membrane (cardiolipin is exposed to the outer membrane as described below). It remains uncertain whether the CDP-DAG produced by ER enzymes is transported to the inner mitochondrial membrane.

In mammalian cells, PS is predominantly synthesized in the ER by the base-exchange reaction of PS synthases using PC (for PS synthase I) and PE (for PS synthase II) as precursors (PC/PE + l-serine → PS + choline/ethanolamine) (Fig. 1B) (16, 17), while PS synthesis in bacteria and fungi occurs by a CDP-DAG pathway (CDP-DAG + l-serine → PS + CMP) (9). The decarboxylation of PS in mitochondria is also an important pathway of de novo synthesis of PE in mammalian cells (18). PE produced in mitochondria is likely transported to the ER (18).

Ceramide, which is the common intermediate for the biosynthesis of SM and glycosphingolipids, is synthesized in the ER (Fig. 1) (19). Ceramide is delivered from the ER to the luminal side of the Golgi apparatus and converted to SM by SM synthase (SMS), which catalyzes the transfer of phosphocholine from PC to ceramide. The human genome contains genes for two different SMSs, SMS1 and SMS2. SMS1, which is mainly responsible for the de novo synthesis of SM, is localized to the *medial*/*trans* Golgi region, while SMS2 resides at the PM as well as the Golgi apparatus and plays a key role in the resynthesis of SM from ceramide generated at the PM. SM is ubiquitous in vertebrates, accounting for 5–10% of all phospholipids (20, 21). Ceramide is also converted to glucosylceramide (GlcCer) by GlcCer synthase (catalyzing the transfer of glucose from UDP-glucose to ceramide). The mammalian genome possesses one gene (*UGCG*) encoding GlcCer synthase, which is mainly distributed to the *cis*/*medial* Golgi region. After being transported to the luminal side of the Golgi apparatus, GlcCer is converted to more complex glycosphingolipids.

Sterols are also ubiquitous in eukaryotes, and the predominant sterol in mammalian cells is cholesterol. De novo synthesis of cholesterol specifically occurs in the ER, because all enzymes dedicated to the biosynthesis of cholesterol localize to the ER (22, 23). Nevertheless, the level of cholesterol in the ER is regulated to be low, while that of the PM is high, indicating that cholesterol is somehow moved from the ER to the PM against its concentration gradient (see below). In the synthesis of steroid hormones, cholesterol is delivered to mitochondria to be oxygenated (24). Although cholesterol is converted to bile acids in the ER in hepatocytes, it is also delivered to mitochondria for the alternative pathway of bile acid synthesis (24).

## THREE LINES OF EARLY RESEARCH THAT LED TO THE CONCEPT OF LTP-MEDIATED INTER-ORGANELLE TRAFFICKING OF LIPIDS AT MCSs

There are at least three lines of early research that led to the concept of LTP-mediated inter-organelle trafficking of lipids at MCSs. By the late twentieth century, it had already been established that secretory proteins and integral membrane proteins synthesized at the ER are delivered to the PM through the Golgi apparatus mainly by membrane-bound carriers named “transport vesicles” in eukaryotic cells (25, 26). Because the ER is the main site for the synthesis of various membrane lipid types, transport vesicles were also expected to play a central role in the inter-organelle transport of lipids synthesized in the ER. However, a number of studies suggested that the inter-organelle transport of lipids occurred by a mechanism(s) that differed from the transport vesicle-mediated pathway. For instance, pharmacological tools and low temperatures that inhibit the ER-to-PM (via the Golgi complex) transport of proteins were shown not to compromise the delivery of PC or cholesterol from cytoplasmic compartments to the PM in mammalian cells (27, 28). Moreover, in spite of the shut off of the ER-to-Golgi vesicular transport pathway in mitosis, de novo synthesized ceramide was shown to be converted to complex sphingolipids (29), indicating that lipids are transported via pathways distinct from the well-known vesicular pathway.

Another important line of research is the recognition of MCSs: MCSs are organelle subregions, in which different organelle membranes come into close apposition (10–30 nm) (7, 8, 30, 31). The close proximity of different organelle membranes was observed in the early era of cell biology by classical electron microscopy (32, 33), and more detailed images of contact structures were later obtained by 3D image reconstruction cryo-electron tomography (34). The description of an ER subregion entity as a membrane fraction associated with mitochondria (“MAM”) was also a milestone for the molecular dissection of ER-mitochondria MCSs (35). However, definitive evidence to show that MCSs are involved in the inter-organelle transport of lipids was not obtained for a long time.

The third line of crucial research is the discovery of LTPs. As symbolized in the nomenclature, LTPs have been defined as proteins capable of transferring lipids between different membranes. Although biochemical studies on LTPs started in the late 1960s using in vitro assay systems (36), it took many years to demonstrate that LTPs play crucial roles in the inter-organelle transport of their lipidic ligands. Various studies demonstrated that some LTPs are not essential for transport events that are anticipated to occur during the metabolism of specific lipid types in cells (37). This may be due to functional redundancy or, alternatively, to previous studies focusing on relatively abundant LTPs, the physiological functions of which may be “cytosolic lipid holders” rather than “inter-membrane lipid carriers”. Nevertheless, mutations in the human gene encoding the steroidogenic acute regulatory protein (StAR), a sterol-transfer protein, were shown to be causative of some types of inherited disorders in steroid hormone production, providing an excellent example for the relevance of in vitro lipid-transfer activity in in vivo functions (38–40). StAR has recently been proposed to act as a subunit of a multi-component complex that enables sterols to traverse the outer mitochondrial membrane, and not as a simple inter-membrane sterol carrier (41). The development of fluorescent analogs of lipids was also a great epoch to facilitate research on the intracellular trafficking of lipids. Optical observations of cell-associated fluorescent lipids have provided insights into the dynamics of lipid transport in intact cells (42); however, the extent to which the behavior of these analogs reflects that of their natural counterparts in cells remains unclear (43–45).

In the early 2000s, a molecular entity that mediates the inter-organelle transport of ceramide was identified after a functional rescue cloning method (46). CERT has biochemical and structural characteristics not only to catalyze the inter-membrane transfer of a lipid, but also to act at ER-Golgi MCSs, providing an entity-based model in which LTPs mediate the inter-organelle transport of lipids at organelle MCSs in a nonvesicular manner (46–48) (see also below).

## CERT AND ITS FUNCTIONAL DOMAINS AND MOTIFS

**Fig. 2.**
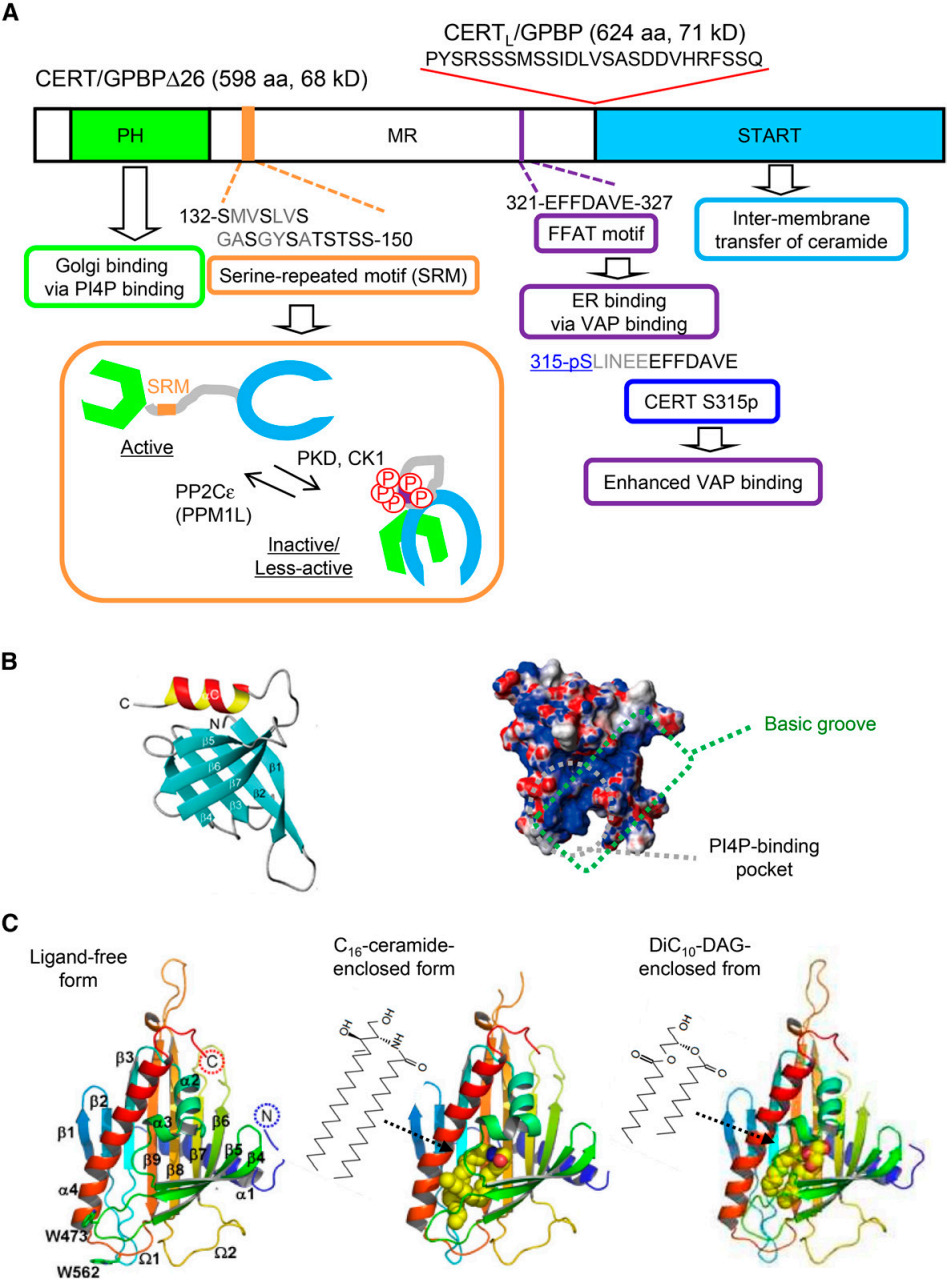
CERT consists of various functional modules. A: The functions of the domains and motifs in human CERT are shown. For detailed functions of the respective domains and motifs, please refer to the text. The splicing isoform CERT_L_/GPBP contains the 26 amino acid residues in front of the START domain. Inset: Downregulation of CERT by hyper-phosphorylation of the SRM. PKD, protein kinase D; CK1, casein kinase 1; PP2Cε (or PPM1L), protein phosphatase 2Cε (or protein phosphatase Mg^2+^- or Mn^2+^-dependent 1L). B: Structure of the CERT PH domain. The 3D structure of the CERT PH domain elucidated by solution NMR spectroscopy is shown as a ribbon model (left panel) and an electrostatic potential map (right panel) (56). The PI4P-binding pocket and basic groove participating in mild binding to acidic phospholipids are indicated (56). C: Structure of the CERT START domain. The 3D structures of the CERT START domain in ligand-free and lipid-enclosed forms elucidated by X-ray crystallography (66) are shown.

Among Chinese hamster ovary cell mutants defective in the synthesis of SM (49), one variant, named the LY-A cell line, was found to have a deficiency in the transport of ceramide from the ER to the Golgi apparatus in spite of no discernible deficiency in ER-to-Golgi transport of membrane proteins (44, 50). After the screening of cDNA that may confer phenotypic recovery to LY-A cells, a human cDNA encoding a cytosolic protein with 598 amino acid residues (named CERT after ceramide trafficking) was identified (46). Accordingly, the gene encoding CERT (as well as its splicing variant with an extra 26-amino acid sequence, as shown in **Fig. 2A**) was named *CERT* (or *CERT*/*CERTL*). The *CERT*/*CERTL* gene of LY-A cells was shown to have a missense mutation, which causes a dysfunction in CERT (46). CERT is identical to a splicing variant of Goodpasture antigen-binding protein (GPBP) (46, 51, 52). The human genomic gene encoding CERT/GPBPΔ26 (and CERT_L_/GPBP) is also referred to as *COL4A3BP* (51, 52). CERT is composed of several functional domains and motifs (Fig. 2).

### Pleckstrin homology domain for association with the Golgi apparatus

The N-terminal region consisting of ∼100 amino acids forms a pleckstrin homology (PH) domain (Fig. 2A), the well-elucidated function of which is phosphoinositide-binding (53, 54). The PH domain of CERT preferentially binds to PI 4-monophosphate (PI4P) among the various phosphoinositides (46, 55, 56). PI4P is mainly distributed to the PM, Golgi apparatus, *trans* Golgi network, and late endosomes/lysosomes (LE/LYs), and serves not only as the main precursor for PI(4,5)-bisphosphate [PI(4,5)P_2_] but also as an organelle-selective functional module by itself (14, 15). The intracellular distribution of phosphoinositides is not stringent enough to explain the organelle-targeting specificity of PH domains only by their phosphoinositide-binding specificity. The PH domain of CERT is structurally similar to other Golgi-targeting and PI4P-preferential PH domains of oxysterol-binding protein (OSBP) and four-phosphate-adaptor protein 1 (FAPP1). The latter two PH domains were shown to use ARF1, a small GTP-binding protein, as a coordinating factor for their specific targeting at the Golgi apparatus (55, 57), and a restricted region of the FAPP1 PH domain surface was shown to interact with GTP-bound ARF1 (58). In contrast, no analogous coordinating proteins for the CERT PH domain have been identified to date (see below).

The 3D structure of the CERT PH domain has been solved by solution NMR spectroscopy (56) and X-ray crystallography (59). The CERT PH domain has two types of binding regions: a conventional phosphoinositide-binding pocket and a previously unrecognized region named a basic groove (Fig. 2B) (56). The basic groove mildly interacts with phospholipid bilayers containing acidic lipids, such as PS, even without PI4P (56). The additive effects of the two regions may create a high affinity of the CERT PH for PI4P-embedded phospholipid membranes, such as the Golgi membrane (56). Various other PH domains also employ lipid cooperativity for their recruitment to specific organelles (54, 60). It is important to note that full-size CERT/GPBPΔ26 (as well as CERT_L_/GPBP) acts as a homo-oligomer (possibly a trimer) (51, 52, 61), suggesting that the multivalent state of the PH domain in one CERT oligomer enhances the effective affinity of CERT for Golgi membranes.

### START domain for the inter-membrane transfer of ceramide

The C-terminal ∼210-amino acid region of CERT forms a StAR-related lipid-transfer (START) domain (Fig. 2A). START domains are a family of LTDs, which were initially recognized by amino acid sequence similarities to the sterol transfer protein, StAR (“START” was named after StAR-related lipid transfer) (Table 1) (62). The human genome encodes 15 START domains, and different START domains have various lipid substrate specificities, but have mutual similarities not only in their amino acid sequences but also in their 3D structures (63, 64). The CERT START domain flexibly recognizes various ceramide species, including dihydroceramide and phytoceramide (46, 65). In cocrystals of the CERT START domain in complex with a long-chain natural ceramide (C_16_-ceramide), one lipid molecule is buried in a long amphiphilic cavity and, at the far end of the cavity, the amide and hydroxyl groups of ceramide form a hydrogen-bonding network with specific amino acid residues (Fig. 2C) (66). In the amphipathic cavity, there is limited space around the C1 hydroxyl group of the ceramide molecule in the cocrystals (Fig. 2C), which indicates that it is spatially impossible to accommodate complex sphingolipids (such as SM and glycosphingolipids) with a bulky head group in the cavity (66). Moreover, different sets of amino acid residues in the cavity participate in hydrophobic interactions with the different types of hydrocarbon chains of ceramides (66).

Although CERT has no discernible activity to catalyze the inter-membrane transfer of sphingosine, SM, or cholesterol, it has discernible activities to extract and transfer DAG, which structurally resembles ceramide, in cell-free assays (46, 65). In previous studies (46, 65), 1,2-dioleoyl-*rac*-glycerol (which is rare or absent as a natural DAG) was used as the radioactive DAG material for lipid extraction and transfer assays because only this subspecies was commercially available. The superior efficiency of CERT-mediated DAG transfer may have been detected if more natural DAG subspecies were used. A cocrystal of the CERT START domain in complex with 1,2-didecanoyl-*sn*-glycerol (diC_10_-DAG), a short-chain unnatural DAG, was obtained (Fig. 2C), indicating that the CERT START domain has a potential to form a hydrogen-bonding network with the polar moiety of DAG accommodated in the hydrophobic cavity (66).

### FFAT motif for associating with the ER

**Fig. 3.**
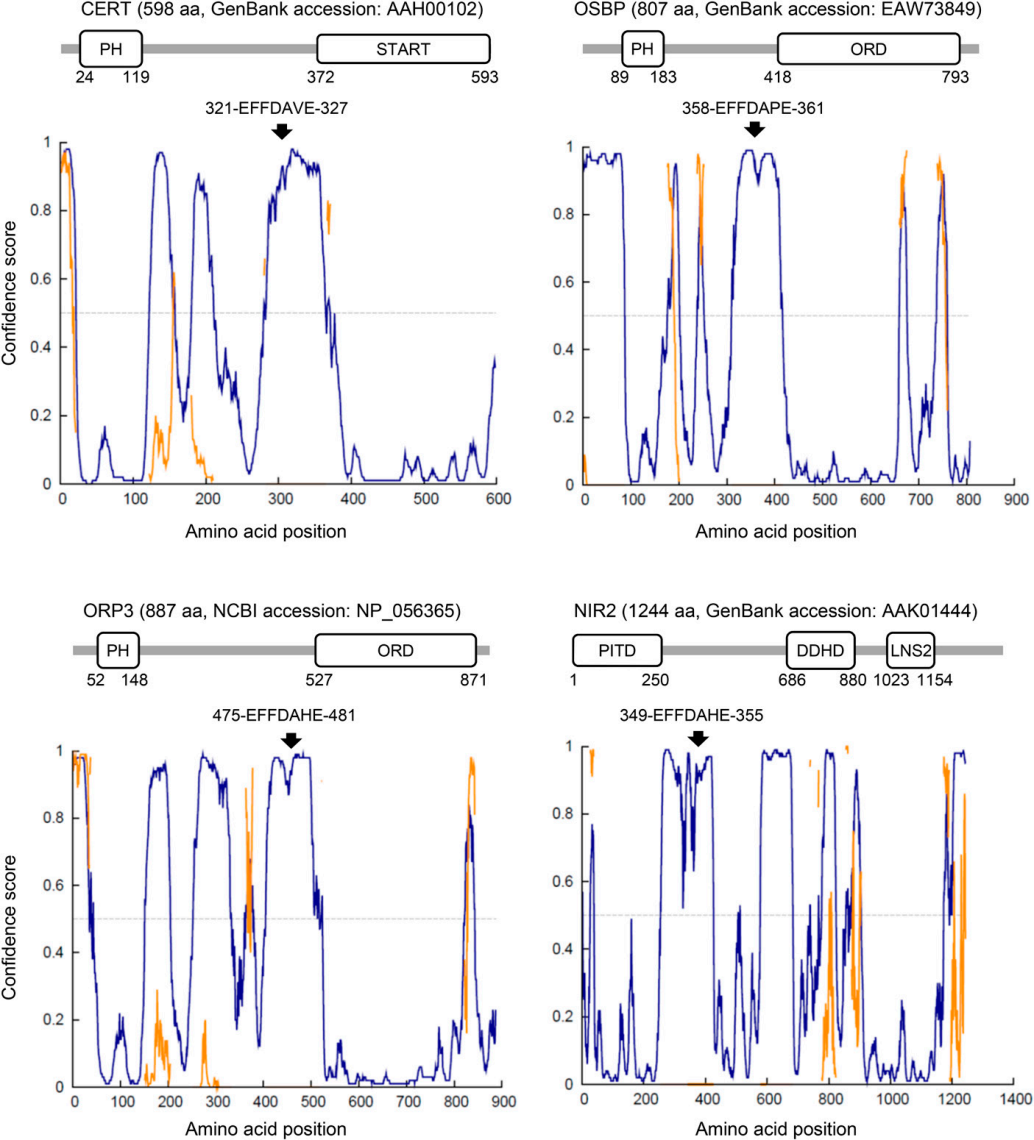
Canonical FFAT motifs localize in disordered regions between structured domains in LTPs. Intrinsic disorder profiles in LTPs with canonical FFAT motifs were analyzed using the DISOPRED3 program (http://bioinf.cs.ucl.ac.uk/psipred/?disopred=1), while domains in the LTPs were analyzed using the SMART program (http://smart.embl-heidelberg.de/) using amino acid sequences from the databank accession number as queries. The start and end amino acid positions of FFAT motifs and globular domains in the proteins are specified in each panel. Nir2 is also known as membrane-associated PITP1 (PITPNM1). Note that the LNS2 domain in human Nir2 was assigned to residues 980–1115 in a previous study (104), while it was assigned to residues 1023–1154 in the search with the SMART program. In addition, a DAG-binding-like segment identified in the previous study (104) was not found in the search with the SMART program. PITD, PI-transfer domain. In the intrinsic disorder profiles: blue line, region with a disordered state; orange line, region with protein-binding activity; arrow, amino acid position of canonical FFAT motifs. FFAT motifs in LTPs localize to disordered regions between structured domains. The motif location may restrict the structured domains within a limited distance from the FFAT/VAP-mediated ER contact sites with appropriate topology.

The middle region (amino acid residues 118–370; hereafter referred to as MR) between the PH and START domains in CERT is predicted to be largely unstructured (or “intrinsically disordered”) (21). The functional importance of disordered regions has been recognized (67, 68): For example, a disordered region of a protein may spatially extend more than a compactly structured domain, thereby enabling its adjacent functional module to catch its ligand molecules that are distributed in a large space. The MR has several modules that associate with or are modulated by other molecules. One of these modules is a short peptidic motif named “two phenylalanines in an acidic tract” (FFAT), which associates with VAP (vesicle-associated-membrane protein-associated protein or VAMP-associating protein), an ER-resident membrane protein (Fig. 2A and **Fig. 3**) (69). Both of the VAP isoforms, VAP-A and VAP-B, appear to serve as an ER-binding partner for various LTPs and other protein families with FFAT or FFAT-like motifs (70, 71). The CERT FFAT motif (321-EFFDAVE-327), which matches the canonical consensus sequence of FFAT motifs (EFFDAxE), has been shown to interact with VAP-A and VAP-B and is crucial for the function of CERT in the ER-to-Golgi trafficking of ceramide (48). Importantly, CERT is capable of simultaneously associating with PI4P-embedding membranes and VAP-residing membranes (48, 72), indicating that CERT may exert its function at ER-Golgi MCSs.

The canonical FFAT motifs in LTPs are often located in disordered regions between structured domains in proteins (Fig. 3). Although the physiological meaning of this currently remains unclear, it is conceivable that the FFAT-motif location contributes to spatially restricting the linked structured domains within a limited distance from FFAT/VAP-mediated ER contact sites (8).

### Phosphorylation sites for the functional regulation of CERT

The function of CERT is posttranslationally regulated by phosphorylation. There are at least two phosphoregulatory sites in CERT (Fig. 2A). One of the two sites is a serine-repeated motif (SRM; 132-SMVSLVSGASGYSATSTSS-150, serine/threonine residues in the motif are underlined), which is located downstream of the PH domain (73). Following the phosphorylation of serine 132 by protein kinase D (74), the serine/threonine residues are sequentially phosphorylated by casein kinase 1 (75). Hyper-phosphorylation in the SRM results in the repression of PI4P binding and ceramide transfer, presumably through a conformational rearrangement of CERT toward PH-START inter-domain mutual functional silencing (Fig. 2A, inset) (73). PH-START inter-domain interference is supported by the recent finding that PH and START domains dock at restricted regions of their surfaces in a cocrystal of the two isolated domains (76). The serine/threonine protein phosphatase, 2C*ε* (also known as protein phosphatase Mg^2+^- or Mn^2+^-dependent 1L), dephosphorylates the SRM of CERT (77). A missense mutation in the human *CERT* (or *COL4A3BP*) gene, which replaces serine with leucine at the amino acid 132 position, is a causative mutation of a mental retardation disorder with dominant inheritance (78), indicating that the functional regulation of CERT by SRM phosphorylation is relevant to human health and disease.

Another phosphorylation site in CERT is serine 315, which is located upstream of the FFAT motif in the MR (Fig. 2A) (72, 79). The phosphorylation of serine 315 markedly increases the FFAT motif-dependent affinity of CERT for VAP, thereby enhancing the ER-to-Golgi transport of ceramide (72). The types of kinases and phosphatases involved in the phosphorylation/dephosphorylation of serine 315 have not yet been identified.

When cells were exposed to myriocin/ISP-1, a potent inhibitor of serine palmitoyltransferase, to block the de novo synthesis of sphingolipids, the SRM was phosphorylated less, whereas serine 315 was phosphorylated more toward the full activation of CERT (72). The state of PI4P in the Golgi membrane is another key factor in the regulation of the Golgi targeting of CERT (80–85). Collectively, multiple mechanisms operate to regulate the function of CERT in cells.

## HOW CAN LTPs GENERATE THE UNIDIRECTIONAL INTER-ORGANELLE TRANSPORT OF LIPIDS IN CELLS?

**Fig. 4.**
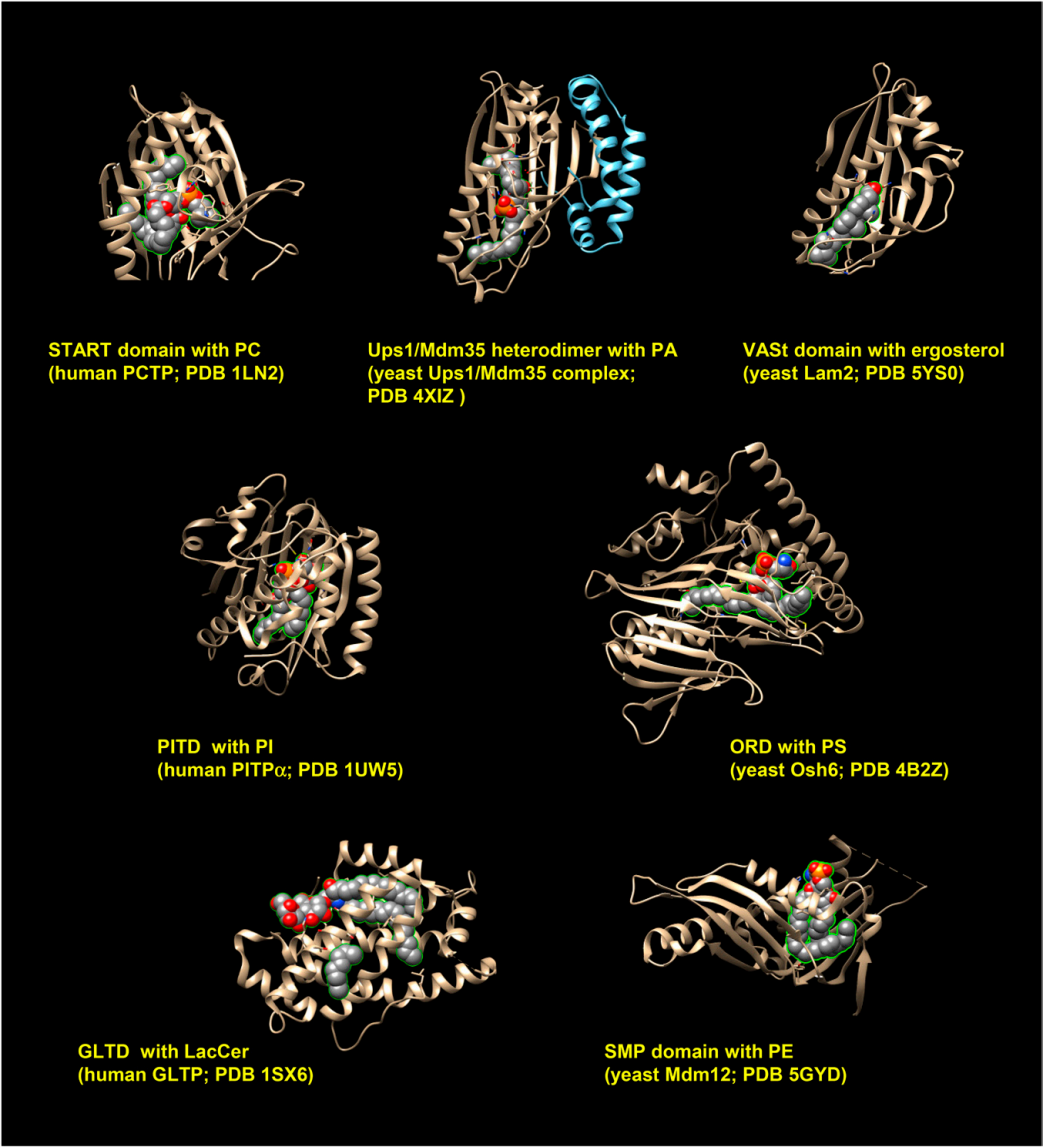
The 3D structures of lipid-enclosed LTDs. X-ray crystallography data of indicated LTDs in complex with lipid ligands was obtained from the RCSB protein database (Protein Data Bank; https://www.rcsb.org/). With the UCSF Chimera program (version 1.12) (225), proteins with ligands were three-dimensionally viewed. Lipid ligands are shown as the sphere model, in which gray, red, orange, and blue spheres represent carbon, oxygen, phosphorus, and nitrogen, respectively. PITD, PI-transfer domain; GLTD, glycolipid-transfer domain; LacCer, lactosylceramide.

Various lipid-binding domains (e.g., PH domain) that serve as functional modules for the recruitment of proteins to specific organelles are capable of associating with a lipid embedded in a membrane mainly via electrostatic interactions between the domain and polar head group of the lipid, but are incapable of extracting the lipid from membranes. By contrast, LTDs are capable of extracting a lipid from membranes by holding the hydrophobic moiety of the lipid molecule in the interior of the domain (**Fig. 4**). To distinguish the two different “lipid-binding” modes, the mode of lipid-binding by LTDs is hereafter referred to as “lipid-enclosing”.

Various LTPs have been shown to mediate the inter-membrane transport of lipids, possibly at MCSs in cells (Table 1). To identify a common feature in LTP-mediated transport systems, four relatively well-characterized systems were selected and are discussed below.

### CERT

**Fig. 5.**
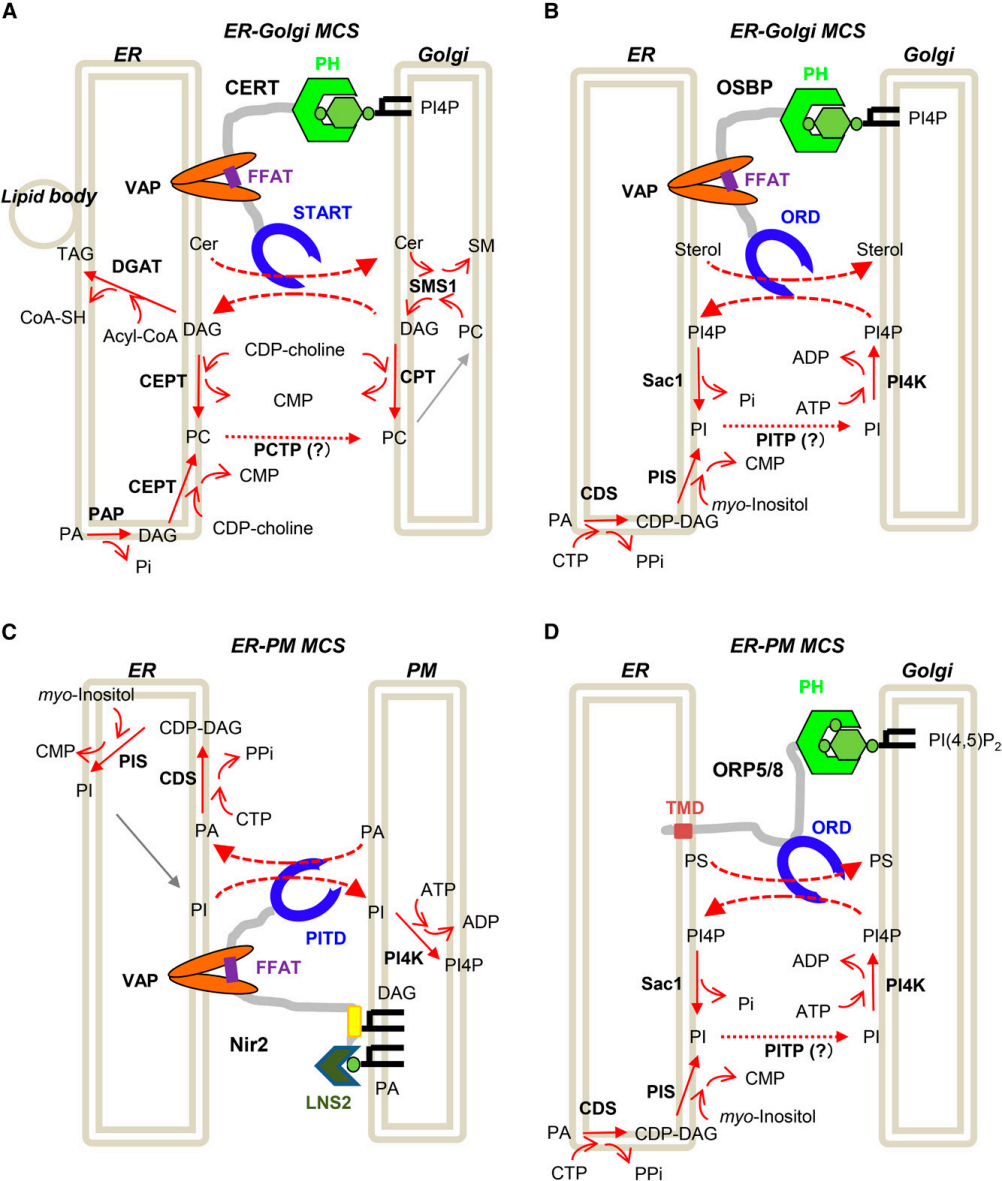
Apparent unidirectional fluxes of the LTP-mediated inter-organelle transport of lipids are indirectly coupled with metabolic reactions of lipids. A: CERT-mediated ceramide/DAG exchange between the ER and Golgi apparatus at MCSs. In this model, CERT binds to the ER VAP (via the FFAT motif) and Golgi PI4P (via the PH domain), extracts newly synthesized ceramide from the ER membrane depending on its START domain, carries the ceramide molecule to the Golgi apparatus at ER-Golgi MCSs, and releases ceramide at the Golgi membrane. On its return, the empty START domain extracts DAG produced during the synthesis of SM and carries the DAG molecule to the ER membrane. DAG may be converted to other anabolic metabolites, such as PC and triacylglycerol. PC serves as a precursor of SM; however, the mechanisms by which PC is transported from the ER to the Golgi apparatus currently remain unclear. DAG produced in the Golgi apparatus may be converted to PC by Golgi-distributed CPT (12). Cer, ceramide; PAP, PA phosphatase; DGAT, DAG acyltransferase; TAG, triacylglycerol. B: OSBP-mediated sterol/PI4P exchange between the ER and Golgi apparatus. In this scenario (99), OSBP bound to two organelles transfers a sterol from the ER to the Golgi apparatus depending on the ORD. In turn, PI4P is transferred from the Golgi apparatus to the ER, and, upon its arrival, PI4P is dephosphorylated to PI by the ER-resident PI4P-phosphatase, Sac1. After being transported to the Golgi apparatus, presumably by a PITP [Nir2 may be at least partly responsible for the ER-to-Golgi transport of PI (81, 106)], PI is converted to PI4P by a Golgi-localizing PI4K. During one cycle of sterol/PI4P exchange between the ER and Golgi membranes, one ATP molecule is consumed, which underlies the energetic basis to drive cholesterol transport against its concentration gradient. The type of PITP responsible for PI transport from the ER to the Golgi apparatus has not yet been identified. CDS, CDP-DAG synthase; PIS, PI synthase; PI4K, PI-4 kinase. C: Nir2-mediated PI/PA exchange between the ER and PM. In this scenario, Nir2 bound to two organelles transfers PI from the ER to the PM, depending on the PI-transfer domain (PITD). In turn, PA is transferred from the PM to the ER, and, upon its arrival, PA is reused for the synthesis of PI in the ER. After being transported to the PM, PI is further anabolized to PI4P and PI(4,5)P_2_. Yellow box, DAG-binding segment. D: ORP-mediated PS/PI4P exchange between the ER and PM. This scenario is analogous to that of the OSBP-mediated pathway. ORP5 has a TMD to reside in the ER and a PI(4,5)P_2_-preferential PH domain to bind to the PM.

The structural and biochemical characteristics of CERT summarized above provided an entity-based model for the LTP-mediated nonvesicular transport of lipids at MCSs (47, 48), as depicted in **Fig. 5A**. When the PH domain and FFAT motif of CERT associate with the Golgi apparatus and ER, respectively, the extraction and transfer of ceramide from the ER to the Golgi apparatus may be attained by “neck-swing”-like movements of the START domain between the two organelles without the cytosolic diffusion of CERT. Under the endogenous expression level of CERT, the PH domain and FFAT motif are both indispensable for supporting the synthesis of SM, while they are dispensable under overexpression conditions (48). In addition, the START domain-only construct exhibits markedly higher activity for the inter-membrane transfer of ceramide than full-size CERT in cell-free systems (46, 73). These findings suggest that CERT has the potential to transfer ceramide from the ER to the Golgi apparatus in a cytosolic diffusion manner, whereas the neck-swing mode at ER-Golgi MCSs plays a major role under physiological conditions (Fig. 5A).

In the biochemical reaction to synthesize SM (ceramide + PC → SM + DAG), DAG is coproduced with SM (Figs. 1, 5A). Because DAG acts as an inhibitor of SMS in vitro (86), the accumulation of DAG at the site of SM synthesis is somehow prevented to continue the synthesis of SM. Whereas de novo synthesis of PC largely occurs in the ER by the catalytic reaction of CEPT, CPT might contribute to PC synthesis at the Golgi apparatus (see the Structure and Biosynthetic Pathways of Major Lipid Types in Mammalian Cells section). Although the fate of DAG produced in the Golgi apparatus is unclear, one possibility is that DAG is converted to PC by CPT in the Golgi apparatus (Fig. 5A). Alternatively, CERT may transfer DAG from the Golgi apparatus to the ER. In the ER, DAG may be converted to PC by CEPT (Fig. 5A). If the latter possibility is the case, the CERT-mediated ceramide/DAG exchange between the ER and Golgi apparatus may construct a recycling system in the cellular lipidome. Although CERT was demonstrated to recognize DAG in addition to ceramide in vitro (46, 65, 66), further studies are needed to prove the physiological relevance of CERT-mediated DAG transfer. Because the catalytic site of SMS is oriented to the luminal side in Golgi membrane bilayers (87), ceramide and DAG have to transverse the Golgi membrane to be moved between CERT and SMS. Although this issue remains elusive, the spontaneous transbilayer movement of ceramide and DAG may be sufficiently rapid to sustain the normal lipidome of cells (88–90).

### OSBP

OSBP, which was initially identified as a cytosolic protein capable of binding various oxysterols (91, 92), is a sterol-transfer protein. OSBP has a PI4P-binding PH domain and canonical FFAT motif, both of which structurally resemble those of CERT (Fig. 3); while the LTD of OSBP [OSBP-related domain (ORD)] structurally differs from the START domain of CERT. OSBP-related proteins (ORPs) in humans and Osh proteins in yeast are members of a family of LTPs with ORDs as their LTDs (93, 94). The yeast, Osh4p (also known as Kes1p), which contains no discernible domains other than the ORD, was crystalized in complex with sterols (95), in line with its in vivo function of ergosterol transfer from the ER to the PM in yeast cells (96). Osh4p was subsequently found to be unexpectedly capable of accommodating PI4P in place of sterols (97), and this PI4P-enclosing feature was demonstrated in other ORDs (98–101).

In CERT-mediated ceramide transport from the ER to the Golgi apparatus, ceramide moves in the direction of its downhill gradient because ceramide is converted to SM upon its arrival at the Golgi apparatus (Fig. 5A). In contrast, several types of lipids are transported in an uphill direction in cells. For example, although the site of de novo synthesis of cholesterol is the ER, the steady state level of cholesterol is low in the ER, but high in the PM. Thus, de novo synthesized cholesterol is moved from the ER to the PM against its concentration gradient. One mechanism underlying this uphill movement is OSBP- or Osh4p-mediated lipid exchange between different organelles with the indirect metabolic coupling of the dephosphorylation and resynthesis of PI4P, through which one ATP is consumed in one complete reaction cycle (Fig. 5B) (97, 99). ORD-mediated PI4P movement from PI4P-synthesizing organelles (e.g., PM and Golgi apparatus) to the ER is along a downhill concentration gradient because PI4P is degraded to PI by the ER-resident PI4P phosphatase, Sac1 (102). The ability to exchange PI4P with a “forward transported” lipid has not been shown in non-ORD LTP (although a START-like domain was recently demonstrated to catalyze inter-membrane transfer of sterols and PI(4,5)P_2_, as described in the VASt proteins section).

### N-terminal domain-interacting receptor 2

N-terminal domain-interacting receptor (Nir)2 (RdgBα in the fly *Drosophila*) is an LTP with a canonical FFAT motif (Fig. 3). The human genome encodes three Nir isoforms: Nir1, Nir2, and Nir3 (with gene names *PITPNM3*, *PITPNM1*, and *PITPNM2*, respectively). Nir2 and Nir3 have an LTD in the N-terminal region as well as a canonical FFAT motif (Fig. 3), while Nir1 has the FFAT motif, but lacks the LTD (103). The LTD of Nir2/3 is a member of the PI-transfer domain family, the structural prototype of which is the PI-transfer protein (PITP)-α isoform (PITPα) (encoded by the *PITPNA* gene) (Fig. 4) (104). PITPα is ubiquitous in vertebrates, and purified PITPα catalyzes the inter-membrane transfer of PI and PC in vitro (105). Although Nir2 was previously suggested to exchange PI and PC between the ER and Golgi apparatus (81, 106), recent studies have shown that Nir2 (and Nir3) exchanges PC and PA between the ER and PM (104, 107, 108). In the latter case (Fig. 5C), Nir2 bound to the two organelles transfers PI from the ER to the PM, depending on its LTD. In turn, PA is transferred from the PM to the ER, and, upon its arrival, PA can be reused for the synthesis of PI in the ER. After being transported to the PM, PI is further anabolized to PI4P and PI(4,5)P_2_.

Nir2 relocates from the perinuclear Golgi region to the PM in response to growth factor stimulation (104). For the relocation of Nir2 to the PM, both the C-terminal Lipin/Ned1/Smp2 (LNS2) domain, which is capable of binding PA, and its adjacent segment, which is capable of binding DAG, are required (Fig. 5C) (104, 107). Nir2/3 may exert dual functions in cells: PI/PC exchange at ER-Golgi MCSs and PI/PA exchange at ER-PM MCSs.

### ORP5 and ORP8

PS is a type of lipid enriched in the cytoplasmic leaflet of PM phospholipid bilayers (1). After being synthesized in the ER, PS is transported to the PM against its gradient. ORP5 and ORP8 in humans (Osh6 and Osh7 in yeast) are at least partly responsible for the uphill movement of PS (100, 101), employing a mechanism that is analogous to the OSBP-mediated sterol/PI4P coexchange (Fig. 5B, D). ORP5and ORP8 have a PI(4,5)P_2_-binding PH domain and an ORD that encloses PS (but not sterol) and PI4P (Fig. 4) (109). These ORPs have no canonical FFAT motifs, but have a transmembrane domain (TMD); therefore, they act as ER-tethered LTPs with a PM-binding module (Fig. 5D). These ORPs are capable of coexchanging PS with PI4P at ER-PM MCSs (Fig. 5D). Although a PH domain-deleted, but PM-linkable, ORP5 mutant construct may mediate uphill PS movements, the inhibition of PI4P metabolism impairs the uphill movements mediated by the mutant ORP5 (101), indicating that coupling with the metabolism of PI4P is required for ORP5-mediated uphill movements of PS. A previous study argued that ORP5 and ORP8 exchange PS with PI(4,5)P_2_, rather than PI4P (110).

The N-terminal regions of ORP5 and ORP8, which contain a polybasic motif and PH domain, serve as a PM-targeting module (111). The PM-targeting module in the two ORPs is capable of binding PI4P and PI(4,5)P_2_, and the ORP5/8-mediated flux of PI4P from the PM to the ER is dependent on the amounts of phosphoinositides in the PM, allowing the ORP5 and ORP 8 systems to act as a rheostat to control the levels of PM PI4P and PI(4,5)P_2_ (111). A similar rheostat function may be archived by OSBP for the level of PI4P in the Golgi apparatus (83, 84).

In addition to ER-PM MCSs, ORP5 and ORP8 also localize to ER-mitochondrial MCSs, in which the ORDs of ORP5 and ORP8 physically interact with the outer mitochondrial membrane protein, PTPIP51 (112). Deletion of ORP5 and ORP8 impairs the morphology and respiratory function of mitochondria (112), indicating a crucial role of ORP5 and ORP8 in mitochondrial physiology. However, effects of the ORP5/8 deletion on the mitochondrial lipidome have not been elucidated. In addition, identification of a mitochondrial PI-4-kinase will be needed to extrapolate the PS/PI4P coexchange mechanism to the ORP5/8-mediated mitochondrial pathway. Most members of the ORP/Osh family (including ORP5/8 and Osh6/7, capable of PS/PI4P-exchanging) exhibit sterol/oxysterol-binding or -enclosing activity (113–116). Furthermore, Osh4 was recently shown to mediate sterol transport from the ER to mitochondria in yeast (116). Thus, an alternative possibility may be that ORP5 and ORP8 facilitate sterol transfer between the ER and mitochondria, along with its chemical gradient, without PI4P coexchange. Notably, cocrystallography of Osh4 in complex with sterols revealed that there is no direct hydrogen-bonding between the hydroxyl group of sterol and amino acid residues of the protein, and that several water molecules mediate interactions between the protein and its lipid-ligand in the hydrophobic cavity (95), which may endow the ORD with a flexibility to accommodate lipid types structurally dissimilar to sterols (113).

### LTP systems may rectify inter-organelle fluxes of lipids by indirect coupling with metabolic reactions

The inter-membrane lipid-transfer reaction (and lipid-exchanging reaction) catalyzed by LTDs is essentially an equilibrium reaction: when an LTP is added to a system with multiple membranes having different concentrations of lipid, the LTP catalyzes the inter-membrane transfer of its ligand lipid toward the equilibrium state, in which the concentrations (more precisely, chemical activities; see the Thermodynamic Nonequilibrium States May Underlie the Enrichment of Cholesterol in the PM section) of the lipid in different membranes are equal. This is the same in the case of the inter-membrane exchange of two lipid types by an LTP (117). Thus, to achieve the transport of lipids toward thermodynamically unfavored directions, additional factors are required. The four systems shown in Fig. 5 have a common mechanistic feature: These LTPs rectify lipid fluxes between different organelles by indirectly coupling with the metabolic reactions that occur in specific organelles (Fig. 5A–D). Even in the case of apparently downhill movements, the anabolic conversion of ceramide to SM in the Golgi site drives the unidirectional flow of ceramide in the CERT system, while the conversion of DAG to other anabolites (e.g., PC and triacylglycerol) in the ER may also accelerate the lipid-exchanging transport cycle (Fig. 5A). The reaction catalyzed by SMS (ceramide + PC → SM + DAG) proceeds without the consumption of biological energy (e.g., hydrolysis of the high-energy phosphate bond in ATP). However, PC, a precursor of SM, is synthesized at the expense of biological energy (DAG + CDP-choline → PC + CMP in the Kennedy pathway) (Fig. 1). In the Nir2 pathway (Fig. 5C), the exchange of PI/PA between the ER and PM may be indirectly accompanied by the anabolic alteration of PI (PI + ATP → PI4P + ADP) in the PM and that of PA (PA + CTP → CDP-DAG + pyrophosphate) in the ER. CDP-DAG may be further converted to PI (CDP-DAG + *myo*-inositol → PI + CMP), thereby refilling the PI pool of the ER (85). The Nir2-dependent system may rectify the direction of the PI flux from the ER to the PM, coupling with the metabolic reactions that occur in different organelles. Therefore, a potential principle is that LTP-mediated inter-organelle lipid transport systems rectify lipid fluxes by indirectly coupling with the metabolic reactions that occur in specific organelles in cells.

## OTHER LTP-MEDIATED PATHWAYS THAT ACT AT MCSs

In addition to START domains, several LTDs have START domain-like 3D folds (118) (Fig. 4). The START domain-like superfamily of LTDs, which has been proposed to be referred to as StARkin (119), contains five family members in eukaryotes: START, VASt (or Lam/Yap/Ltc; see below), PITP, Bet_v1 (the major birch pollen allergen), and proteins of relevant evolutionary and lymphoid interest (PRELI) [PRELI-like domain (PRELID)]. Intriguingly, the 3D structure of the human PRELID1/TRIAP1 (and yeast Ups1/Mdm35) heterodimer is similar to that of other StARkin members, which may function in monomeric states (120–122) (Fig. 4). It is likely that different StARkin members without discernible amino acid sequence similarities have convergently evolved to form the 3D fold for their lipid-enclosing function. The applicability of the proposal in the section, LTP systems may rectify inter-organelle fluxes of lipids by indirect coupling with metabolic reactions, to several other LTP systems is described below.

### STARD7, the PRELID/TRIAP complex (or yeast Ups/Mdm35 complex), and StAR

Mitochondria are incapable of synthesizing PC and, thus, have to gain PC from the ER (4, 5). STARD7, a mitochondrion-tethered LTP, is responsible for the PC acquisition of mitochondria (123). The STARD7-mediated transport of PC from the ER to mitochondria is downhill, allowing its apparent unidirectional flux without any metabolic alterations in PC in mitochondria. In LTP-mediated intra-mitochondrial transport pathways (i.e., between the outer and inner membranes in mitochondria), several glycerophospholipid types are coupled with their metabolic alterations. For example, after being transported from the ER to mitochondria, PS is converted to PE by the action of PS decarboxylase localized to the inner membrane of mitochondria, while PA is converted to PG and cardiolipin by mitochondrion-localized enzymes (Fig. 1) (4, 5). LTPs that have been proven to be involved in the intra-mitochondrial pathways include: complexes of PRELID1 (Ups1 in yeast) with TRIAP1 (Mdm35 in yeast) for PA (120–122, 124–126) and complexes of PRELID2 (Ups2) with TRIAP1 (Mdm35) for PS (Table 1, Fig. 4) (125, 127, 128). The StAR protein mediates the transport of cholesterol from the ER to mitochondria and/or from the outer to the inner membranes in mitochondria for oxidative alterations in cholesterol to steroid hormones (Table 1) (40, 41, 129), while it is unclear how steroid hormones are released from the mitochondria following their synthesis (e.g., by spontaneous diffusion or a protein-mediated transport pathway). There is the possibility that multiple sterol-transfer proteins (including proteins that lack any domains and organelle-targeting modules outside of sterol-transfer domains) are involved in the sterol fluxes from the ER to mitochondria and also from mitochondria to other organelles (22, 41).

### Glycolipid-transfer proteins

The LTD found in mammalian glycolipid-transfer proteins (GLTPs) is referred to as the glycolipid-transfer domain (Table 1, Fig. 4) (130–132). FAPP2 with a glycolipid-transfer domain may mediate GlcCer from the *cis*-Golgi to the *trans*-Golgi regions (133, 134) or, alternatively, from the *cis*-Golgi to the ER (Table 1) (45). In the *trans*-Golgi region, GlcCer undergoes an anabolic conversion (GlcCer + UDP-galactose → lactosylceramide + UDP) (Fig. 1). In the alternative route (25), after being transported from the *cis*-Golgi to the ER, GlcCer may be sorted to the conventional vesicular transport pathway to reach the *trans*-Golgi region. Lipid substrates of “glycolipid”-transfer domains do not appear to be limited to glycolipids: the ceramide-1-phosphate (C1P)-transfer protein (135), which has a glycolipid-transfer domain, transports C1P presumably from the *trans*-Golgi network to the PM (Table 1) (135); however, the metabolic fate of C1P in the PM is unclear.

### VASt proteins

Another recently identified LTD type is the VASt domain, which was named after the vascular associated death 1 analog of StAR-related lipid transfer (Table 1, Fig. 4) (118, 136, 137). A PH domain-like GRAM domain (GRAMD) is a phosphoinositide-binding module (GRAM was named after glucosyltransferases, Rab-like GTPase activators, and myotubularins) (138, 139). Human GRAMD proteins have three subclasses: GRAMD1 isoforms (1a, 1b, and 1c) have GRAMD at the N-terminal region of the protein, a TMD at the C-terminal region, and a VASt domain at an intervening region; whereas GRAMD2 and GRAMD3 isoforms (2, 2a, 2b, and 3) have GRAMD and TMD, but not a VASt domain. Although the binding partners of GRAMDs have been poorly determined, the domain of GRAMD2a was recently shown to bind PI4P and PI(4,5)P_2_ (140).

The yeast Lam/Yap/Ltc proteins are orthologs of human GRAMD1 isoforms (a few of the yeast isoforms contain two VASt domains in a protein). The yeast Lam/Yap/Ltc proteins mediate sterol transport between the ER and PM (141, 142) and/or between the ER and mitochondria or vacuoles (Table 1) (143, 144). It has recently been shown that the VASt domains of GRAMD1a–c catalyze inter-membrane transfer of sterols and that the domain of GRAMD1b is also capable of transferring PI(4,5)P_2_ in vitro (145) (the in vivo significance of the findings on GRAMD VASt domains has not yet been tested). It currently remains unclear whether VASt protein pathways mediate unidirectional lipid fluxes along downhill gradients or against uphill gradients and whether lipid fluxes couple with the metabolism of lipids. These pathways may be physiologically bi-directional.

### SMP proteins

The synaptotagmin-like mitochondrial-lipid-binding (SMP) domain (SMP is a family of the TULIP superfamily) is a type of LTD (Table 1, Fig. 4) (146, 147). Several SMP proteins are responsible for the inter-organelle transport of glycerophospholipids. One well-characterized SMP protein type is the extended-synaptotagmins (E-Syt1, -2, and -3) [yeast orthologs are tricalbins (Tcb1, -2, and -3)]. Each E-Syt has an ER-spanning TMD at the N-terminal region, which is followed by the SMP domain and multiple C2 domain (the most C-terminal C2 domain capable of binding PI(4,5)P_2_ acts as the PM-binding module), and localize to ER-PM MCSs (148–150). E-Syt2 has been demonstrated to mediate ER-to-PM transport of several types of phospholipids in mammalian cells (151). E-Syt1 and -3 are expected to play a similar role. TMEM24 (also known as C2CD21), an SMP protein with an ER-spanning TMD, was recently found to mediate PI transport between the ER and PM (152).

Another well-characterized type of SMP protein is the ER-mitochondrial MCSs: yeast Mmm1, Mdm12, and Mdm34 (Table 1) (153–156). Monomers, homodimers, or heterodimers of Mmm1, Mdm12, and Mdm34 exhibit inter-membrane phospholipid transfer activity in vitro (153–156). Importantly, they form a hetero-oligomer complex [which is the core entity of the ER-mitochondrial encounter structure (ERMES)] with Mdm10, a non-SMP protein spanning the outer membrane of mitochondria, at ER-mitochondrial MCSs in yeast (157, 158). The ERMES complex is supposed to act as a physiological SMP protein complex that mediates inter-organelle phospholipid transport between the ER and mitochondria at MCSs (153–156). Besides the ER-to-mitochondria transport of phospholipids, the ERMES complex may facilitate the transport of PE and PG produced in mitochondria to the ER, presumably along their downhill gradients.

A recent study revealed that human PDZD8, an ER-mitochondria tethered protein with an SMP domain, is involved in the regulation of mitochondrial Ca^2+^ import upon induction of ER Ca^2+^ release in neurons (159). PDZD8 has no significant extra homology to Mmm1 over other SMPs and has multiple other domains (159). In addition, the heterologous expression of human PDZD8 in yeast cells failed to rescue the phenotype of Mmm1-defecitve cells (159). Thus, it remains unclear whether PDZD8 catalyzes phospholipid-transfer between the ER and mitochondria in mammalian cells; and more studies will be required to convincingly annotate PDZD8 with a human ortholog of Mmm1.

Yeast nuclear membrane-vacuole junction (Nvj)2 is an ER-tethered protein with a PH domain and a SMP domain (160). Nvj2 was initially shown to be localized to the Nvj in yeast, and it was suggested to be involved in sterol transfer between the nuclear envelope and vacuole (160). It was later found that, under ER-stressed conditions, Nvj2 relocates to ER-Golgi MCSs and facilitates transport of ceramide (more precisely, phytoceramide, a ceramide isoform predominant in yeast) from the ER to the *cis*-Golgi region, in which ceramide is converted to the yeast sphingophospholipid, inositol phosphoceramide (IPC) (161). Under normal culture conditions, ER-to-Golgi transport of yeast ceramide is mainly mediated by a vesicular pathway regulated by Osh proteins (162), and IPC synthesis is not affected by disruption of the *Nvj2* gene (161). Although TEX2 (also known as HT008) is likely a human ortholog of Nvj2 (160), its localization and function in mammalian cells are currently unknown. Cholesterol ester-transfer protein is an SMP protein (147, 163), which shuttles various lipid types (e.g., cholesterol ester, triacylglycerol, and PC) between lipoproteins, not organelles, in the blood.

The 3D structure of the lipid-enclosed forms of SMP domains revealed an interesting topological feature in lipid molecules enclosed by the domain (151, 153–156, 163), which differed from the feature of lipid molecules enclosed by other LTD types (Fig. 4). Namely, in the START (66, 164) and VASt (136, 137) domains, the polar head moieties of the enclosed lipids are placed in the interior of the domain, forming hydrogen-bonding networks, while they largely protrude from the surface of SMP domains, with only the hydrophobic acyl chains of the lipids being placed in the interior of the domains (151, 153–156, 163). The different topologies of the enclosed lipid molecules may explain why one type of SMP domain is capable of enclosing lipids with different polar head groups. When SMP domains bridge different organelles, the SMP bridge may function as a “lipid channel” (151, 156) or “lipid slider” (153, 155), rather than a “lipid carrier,” at MCSs. The broad range of lipid recognition by SMP domains may enable the ER to supply other organelles with a housekeeping set of phospholipids (such as PC, PE, PS, and PI, which are ubiquitous components in most organelles). An analogous lipid-sliding mechanism may function in the translocation of endotoxin lipopolysaccharides from the inner to the outer membrane via a membrane-to-membrane bridging complex in the envelope of Gram-negative bacteria (165).

Cardiolipin, a lipid with four acyl groups per molecule, is synthesized in the inner membrane of mitochondria (Fig. 1). Cardiolipin strictly stays in the inner mitochondrial membrane in healthy cells. However, when mitochondria are damaged, cardiolipin is externalized to the surface of the outer membrane, depending on a hexameric intermembrane space protein, NDPK-D (also known as NM23-H4) (166), and the phospholipid, scramlase-3 (167). NDPK-D/NM23-H4 has been suggested to be a dual functional protein acting as a nucleoside diphosphate kinase and a broad-range LTP with no typical LTD-like structure (168, 169). The externalized cardiolipin is recognized by the autophagy protein, LC3, for the elimination of damaged mitochondria by mitophagy (166, 167). Interestingly, cardiolipin appears not to be diffused from the damaged mitochondria to other organelles (166, 167). Neither Mdm12 nor the Mmm1/Mdm12 complex enclose cardiolipin (153, 154, 156), which may be a biochemical reason for the spatial restriction of cardiolipin to mitochondria.

A recent study showed that human GRAMD2a, which does not contain a VASt domain, but not GRAMD1a, colocalizes with E-Syt2/3 at ER-PM MCSs in a PI(4,5)P_2_-dependent manner (140), suggesting functional coordination between the E-Syt and GRAMD2 systems at MCSs.

### LTPs associated with LE/LYs

Niemann-Pick type C (NPC) is an inherited lipid-storage disorder. In NPC cells, the existence of free cholesterol from LE/LYs to the ER is impaired by dysfunctional mutations in the *NPC1* or *NPC2* gene, the products of both of which specifically locate to LE/LYs (170). The NPC2 protein is a soluble LTP inside LE/LYs, and it transfers cholesterol from internalized lipoproteins (especially LDL) to NPC1 (171–174), presumably along with the downhill gradient of cholesterol. NPC1, which has several sterol-sensing domains in its putative 13 TMDs (175), plays an essential role in the egress of cholesterol transverse from LE/LYs. Nevertheless, it has not been determined whether NPC1 is capable of catalyzing transmembrane movement of sterol (176).

Previous studies have suggested that there are at least two pathways to transfer cholesterol from LE/LYs to the ER: approximately 70% of lipoprotein-derived cholesterol is thought to be transported from LE/LYs to the PM and then to the ER (177), while the residual fraction may be delivered directly to the ER (178). However, the molecular entities involved in the transport pathways have been unknown for a long time. Recent studies have revealed that LTPs play key roles in sterol transport between LE/LYs and the ER. STARD3 [also known as metastatic lymph node 64 (MLN64)] is a sterol-recognizing START protein with LE/LY-tethering TMDs and an FFAT-motif (179). STARD3 was recently shown to mediate cholesterol transport from the ER to LEs at ER-LE MCSs at the expense of PM cholesterol (180). In this scenario, when PM cholesterol is fluxed into the ER, STARD3 may relocate ER-accumulating cholesterol to LEs and maintain the low level of ER cholesterol (180). ORP1L is an ORP member that has multiple functional modules, including ankyrin repeats, PH domain, FFAT motif, and ORD (181). A previous study showed that ORP1L acts as a cholesterol-liganded regulator for the formation of ER-LE/LY MCSs (182). A recent study argued that, when the LDL-derived cholesterol level is low, ORP1L-dependent transport of cholesterol from the ER to endosomes is required for the formation of multivesicular bodies in LEs (183). Another recent study showed that ORP1L acts as the primary LTP for cholesterol egress from LE/LYs to the ER at MCSs (184). Collectively, these recent studies indicated that sterol-transfer proteins, such as STARD3 and ORP1L, mediate cholesterol transport between LE/LYs and the ER at their contact sites. Importantly, SM is enriched in LE/LYs compared with the ER, which means that LE/LYs have a higher capacity (per area) of accumulating cholesterol than the ER, as discussed in the section, Thermodynamic Nonequilibrium States May Underlie the Enrichment of Cholesterol in the PM. It may be conceivable that STARD3 and ORPL1 catalyze the relocation of cholesterol between LE/LYs and the ER simply along with the downhill gradient of cholesterol, whereas ORPL1 seems to be able to exert uphill transport toward LE/LYs by the sterol/PI4P coexchange mechanism (184), although it remains elusive how LE/LYs can produce PI4P.

Mammals are unable to synthesize α-tocopherol (vitamin E) and, therefore, have to ingest it from their diet. After absorption at the intestine, α-tocopherol is internalized into the liver via the hepatic portal vein. In hepatocytes, endocytosed α-tocopherol is transported from LE/LYs to the PM and is subsequently released into the extracellular fluid for the whole-body circuit (185). Because the α-tocopherol-transfer protein has the ability to bind PI(4,5)P_2_ (185), α-tocopherol appears to be transferred from LE/LYs to the PM (Table 1), with the direction of the α-tocopherol flux possibly being downhill.

## WHAT IS THE MAJOR ADVANTAGE FOR LTPs TO ACT AT MCSs?

What is the primary advantage for LTPs to act at MCSs? The diffusion formula (*L*^2^/*D*, where *L* is length and *D* is the diffusion coefficient) predicts that the short distance between the start and goal points markedly shortens the time period needed by a material to be delivered in a random walking manner. For example, the time period for calcium ions [*D* in water = ∼1 × 10^−9^ m^2^/s (186)] to traverse the cytosol in a human egg cell (∼100 μm in diameter) is calculated to be ∼10 s, while calcium delivery may be accomplished in ∼10 ns in MCSs (∼10 nm). However, the rapid simple diffusion of hydrophobic molecules between different membranes through the aqueous cytosol rarely occurs, even over a very short distance, because the transfer of a hydrophobic molecule from a hydrophobic environment to an aqueous phase encounters a high thermodynamic energy barrier (187–189). An LTD may catalyze the inter-membrane transfer of a lipid by sequestering the hydrophobic moiety of the lipid molecule into the hydrophobic interior of the protein domain.

Although the primary advantage of inter-organelle lipid transport at MCSs may be to shorten the time periods required for delivery, a thermodynamic evaluation predicts that time advantages may be negligible in small spaces (e.g., a 10–30 nm gap at MCSs) because the rate-determining step in the inter-membrane transport of lipids is largely attributed to the desorption of a lipid from membranes, not the diffusion of a lipid-enclosed LTD (189). In contrast, in markedly larger spaces (e.g., in the cytosol of mammalian cells with a diameter of more than ∼40 μm), a significant advantage in the time period may occur (189). This evaluation has been performed based on a two-compartment model in which a cell only has the ER and PM (as a donor and acceptor, respectively) for simplicity (189); however, a eukaryotic cell is a more complicated compartment system.

**Fig. 6.**
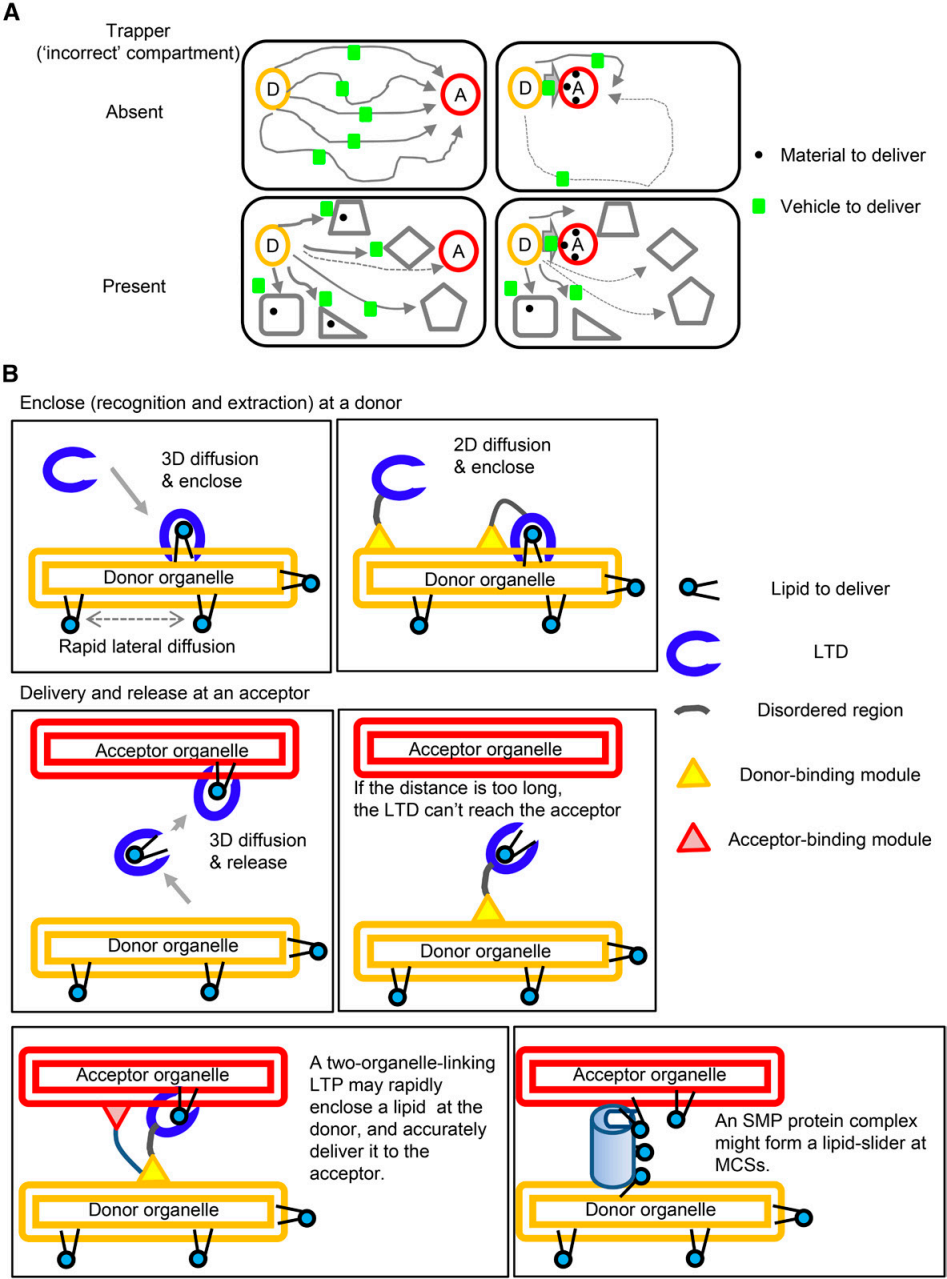
Accurate delivery in the LTP-mediated inter-organelle transport of lipids at MCSs. A: Two systems with different numbers of compartments in the same space volume. A material-carrying vehicle is released from a specific compartment (D, donor) and diffuses in the space in a random-walking manner. The system has only one correct compartment (A, acceptor) to which the material is targeted to reach, while all non-donor compartments are capable of irreversibly trapping the material upon meeting. In a space with only two compartments, a material reaches the acceptor with 100% accuracy, independently of the distance of the two compartments (upper panels). In a space with many compartments (lower panels), accuracy depends on the relative distance among the donor, acceptor, and other compartments. See also the text. B: Mechanistic insight into the advantage of LTP-mediated inter-organelle transport of lipids at MCSs. In the step of enclosing a specific lipid synthesized in a donor organelle, an LTD prebound to the donor organelle may meet the lipid more efficiently than a cytosolically diffusing LTD (upper panels). However, if the donor organelle-bound LTD in complex with the lipid is incapable of reaching the acceptor organelle, the LTD cannot mediate the inter-organelle transport of the lipid (middle panels). This issue is overcome if the donor-bound LTD is capable of simultaneously associating with the acceptor organelle (lower left panel). For an SMP protein complex bridging two organelles, lipid molecules may slide through the complex, inserting their hydrophobic moiety in the interior of the protein and protruding their hydrophilic moiety into aqueous environments (lower right panel).

The efficiency of delivery of a material is affected not only by how rapidly the material is delivered but also by how accurately it is delivered: If a vehicle driver does not know the address of the acceptor, the material will not reach the correct acceptor even with a high-speed vehicle. To consider the accuracy of LTP-mediated lipid transport, two systems with different numbers of compartments are here compared. A material-carrying vehicle is released from a specific compartment (“donor”), and then diffused in the space in a random-walking manner (**Fig. 6A**). The system has the sole correct compartment (“acceptor”), to which the material is desired to reach, while all of the nondonor compartments are capable of irreversibly trapping the material upon meeting with the material-carrying vehicle (e.g., by metabolic conversion). In a space with only two compartments, a material-carrying vehicle released from the donor may finally reach the acceptor with the same 100% accuracy (but with different time periods) independently of the distance of the two compartments (Fig. 6A, upper panels). By contrast, in a space with many compartments, accuracy depends on the relative distance among the donor, acceptor, and other compartments, due to the trapping of the material by intervening “incorrect” compartments (Fig. 6A, lower left panel). Even in the multi-compartment system, when the acceptor is located in close proximity to the donor, the material may reach the acceptor rapidly with high accuracy without being trapped by incorrect compartments (Fig. 6A, lower right panel). Moreover, the misdelivery of materials not only reduces transport efficiency but may also have adverse effects. The redirecting of CERT to mitochondria by swapping its Golgi-targeting PH domain to a mitochondrion-targeting module induces cell death (190), demonstrating that the misdelivery of ceramide may be cytotoxic. Therefore, in addition to speed, accuracy is one of the most essential factors affecting the efficiency of delivery.

To enclose a target lipid, an LTD must meet the lipid, which can diffuse laterally in the membrane. When an LTD encloses a specific lipid synthesized in a donor organelle, an LTD stably bound to the donor organelle is expected to meet the lipid more rapidly than a cytosolically diffusing LTD (Fig. 6B, upper panels). However, if the donor organelle-bound LTD is physically incapable of meeting the acceptor organelle, the rapid enclosing of the lipid at the donor will be to no avail for the aim of the inter-organelle transport of the lipid (Fig. 6B, middle panels). By contrast, if the donor-bound LTD is capable of simultaneously binding the acceptor, the transfer of the enclosed lipid to the acceptor may be attained rapidly and accurately (with no delivery to any incorrect compartments) (Fig. 6B, lower left panel). An SMP protein complex might form a lipid-slider at MCSs (Fig. 6B, lower right panel), as discussed above. Overall, the LTP-mediated lipid transport systems depicted in Fig. 5 may create a mechanical basis underlying the apparent uniflow of a lipid from a specific organelle to another organelle with high accuracy with minimum consumption of biological energy.

## THERMODYNAMIC NONEQUILIBRIUM STATES MAY UNDERLIE THE ENRICHMENT OF CHOLESTEROL IN THE PM

**Fig. 7.**
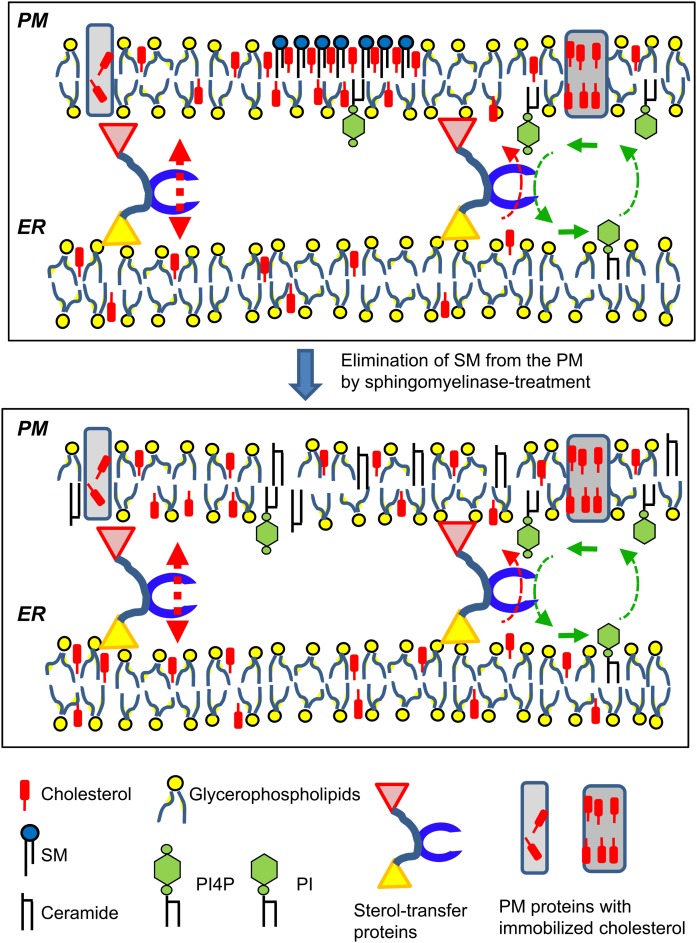
Schematic model of cholesterol enrichment in the PM. The PM has at least three distinct pools of cholesterol (see the text): *i*) distributed to non-raft phospholipid matrix; *ii*) distributed to SM/cholesterol-rafts; and *iii*) immobilized with transmembrane proteins. Cholesterol is exchangeable between pools *i* and *ii*. In contrast, once cholesterol enters pool *iii* (i.e., firmly associates with membrane proteins), it is not free to be exchanged with other pools. In normal cells, the inter-organelle bulk flow of cholesterol synthesized in the ER may proceed with the aid of various sterol-transfer proteins in the downhill direction. Cholesterol can be more concentrated in pool *ii* than in pool *i*, although the chemical activity of cholesterol in the two pools is equal. Note that biological energy is required for the delivery of SM to the PM. Sterol/PI4P-coexchanging transfer systems, which can mediate the uphill movement of cholesterol from the ER to the PM at MCSs, may be responsible for a quantitatively smaller cholesterol flux, although this energy-consuming flux presumably plays an important role in the homeostasis of the minimum requirement of PM cholesterol. Provided that the chemical activity of cholesterol in PM pools *i* and *ii* is also similar to the activity in the ER, the elimination of SM from the PM makes the chemical activity of the bulk cholesterol in the PM higher than that in the ER and, consequently, drives PM cholesterol to move to the ER. Here, inter-membrane transfer of cholesterol may be mediated by sterol-transfer proteins along with chemical activity.

PC and cholesterol are the two most abundant lipid species in mammalian cells (1). Whereas PC is the most abundant phospholipid class in all organelles, cholesterol exhibits highly biased distributions: The PM exhibits a high cholesterol/phospholipid ratio (∼1.0), while the ratio in the ER (in which cholesterol is synthesized) is low (∼0.15) (1). This has been a long-term mystery, and various possibilities to explain the mystery have been proposed. The PI4P-coexchange model has provided a new elegant scenario, as described above. However, this scenario may not be the sole mechanism to generate the biased distribution of cholesterol. Although the uphill movements of cholesterol and PS may be achieved by OSBP- and ORP5/8-mediated pathways (Fig. 5B, D), it currently remains unclear whether the relatively small pool of PI4P actually supports the overall redistribution of more abundant partners (i.e., cholesterol and PS) from the ER to other organelles. All eukaryotes, from yeast to human, seem to have various sterol-transfer proteins: they include multiple ORD, START, and VASt proteins (Table 1). Some of them may act at ER-PM MCSs. Thus, cells may employ multiple pathways for the same lipid type in parallel to sustain the different lipid compositions among different organelles. As for phospholipids, broad-range lipid transfer systems (e.g., SMP protein complex) may mediate a bulk flow of various major phospholipid types from their sites of synthesis to other organelles along with their downhill gradients, while some specific LTP systems (e.g., ORP5/8 for PS) may mediate relatively small fluxes to create the biased distributions of phospholipids at the expense of biological energy (**Fig. 7**). These possibilities are questions to be addressed in the future.

A substance is spontaneously fluxed toward a state with a lower level of free energy (or Gibbs energy) of the system. In contrast, concomitant consumption of energy is required for attaining a thermodynamically unfavored partition of a substance among different compartments in the system. Chemical potential or chemical activity, rather than concentration, of a substance, predicts the tendency of the substance to change its location (191): The chemical potential of a substrate in an ideal solution (infinite diluted solution) is determined by the molar concentration of the substrate, temperature, and pressure (191). In nonideal solutions, interactions between molecules cannot be neglected. Thus, the chemical potential of a substance is corrected with appropriate deviations, and the corrected physical quantity is referred to as chemical activity. When all organelles in a cell undergo the same levels of temperature and pressure, the chemical activity of a lipid species embedded in the membrane of a specific organelle is determined largely by its 2D molar concentration in the membrane and its intermolecular interactions with environmental constituents. Because the cholesterol-interactive phospholipid, SM, is enriched in the PM, it has been proposed that there may be no difference in the chemical activity of cholesterol between the PM and ER in spite of a ∼7-fold difference in the apparent cholesterol concentrations between the two organelles (2, 24, 192–194). However, how SM is enriched in the PM also needs to be considered from a nonequilibrium thermodynamic aspect. I here point out that remote energy-consuming steps may contribute to the enrichment of a specific lipid type in specific organelles. SM newly synthesized in the lumen of the Golgi apparatus is predominantly transported to the PM by a vesicular pathway (195, 196), the processes of which are accompanied by the consumption of biological energy. This may allow SM to be enriched in the PM (more precisely, in the outer leaflet of PM phospholipid bilayers). Cholesterol interacts with SM more preferentially than other lipid types (197–199), and the deprivation of SM from the PM facilitates the relocation or desorption of cholesterol from the PM (200, 201). Thus, the PM pool of SM, which is enriched following energy-consuming events, may serve as a trapper of cholesterol, thereby sustaining the enrichment of cholesterol in the PM (Fig. 7). Other raft constituents, such as glycosphingolipids and PC, with saturated acyl chains may also thermodynamically contribute to the enrichment of cholesterol in the PM (192).

It has recently been revealed that there are three types of cholesterol pools in the PM of mammalian cells (202): *i*) a pool accessible to the cholesterol-binding probe, perfringolysin O (this pool likely represents cholesterol distributed to phospholipid bilayers in the liquid-disorder phase “non-raft membranes”); *ii*) an SM-sequestered pool that binds perfringolysin O only after the enzymatic elimination of SM (this pool is presumably attributable to SM/cholesterol-rafts having liquid-order nature); and *iii*) a residual pool that does not bind perfringolysin O even after SM elimination. Cholesterol released from the SM-sequestered pool is free to translocate to the ER in a nonvesicular manner (202). In contrast, cholesterol of the residual pool (which amounts to ∼10 mol% of total PM lipids) stays in the PM as a minimum requirement to maintain cell viability (202). The apparently immobile property of cholesterol in the third pool is unusual, because lateral diffusion of lipid molecules occurs rapidly in phospholipid bilayers (203). Whereas spontaneous transbilayer movements (“flip-flop”) of polar lipids are slow, those of nonpolar or neutral lipids, including cholesterol, are rapid (89, 204), ruling out the possibility that the immobile fraction is ascribed to the cytosol-oriented fraction of cholesterol in PM bilayers. Although the entity of the third pool remains elusive, it might represent cholesterol molecules immobilized in the PM by direct binding to membrane proteins, or by forming cholesterol-enriched shells around them to exert major regulatory effects on protein function (205). It is also currently unclear whether different transport systems may convey cholesterol to different pools of PM cholesterol. It can be hypothesized, however, that the bulk flow of cholesterol between the ER and PM is mediated by multiple sterol-transfer proteins along with the chemical activity gradient of cholesterol, and that a relatively small amount of cholesterol is actively supplied to the specific PM cholesterol pool essential for cell viability by the sterol/PI4P-exchange mechanism (Fig. 7). Among various organelles, both cholesterol and SM are abundant in LE/LYs next to the PM (1). Thus, LE/LYs may serve as a cholesterol reservoir as described in the section, Other LTP-Mediated Pathways that Act at MCSs.

It should be emphasized that the apparently unidirectional fluxes of lipids and the biased partitioning of lipids among different organelles are sustained at nonequilibrium states in which biological energy is continuously expended. This is in line with the fundamental principle of all dynamic events occurring in living organisms, as pioneeringly stated by physicist Erwin Schrödinger in 1944 (206).

## FUTURE DIRECTIONS

In the past decade, the concept that LTPs play central roles in the inter-organelle transport of lipids at MCSs in cells has been gradually accepted. However, various issues relevant to LTP-mediated lipid trafficking remain unanswered or overlooked. First, even for major lipid types, their inter-organelle transport pathways have not been completely identified. For example, the mechanisms by which the main phospholipid type, PC, is transported from the ER to other organelles remain poorly understood, whereas several LTPs, including SMP domain protein complexes (153, 154, 156) and STARD7 (123), are likely involved in mitochondrial PC pathways. Although the pathway to transport ceramide from the ER to the site for synthesis of GlcCer differs from the CERT-mediated pathway or conventional vesicular pathway (46, 207, 208), how ceramide is transported to the GlcCer synthesis site has not been elucidated. The issues described in the section, Thermodynamic Nonequilibrium States May Underlie the Enrichment of Cholesterol in the PM, also need to be addressed.

Furthermore, the mechanisms by which LTP pathways are regulated have yet to be clarified; only limited types of LTPs are known to be regulated by phosphorylation (**Table 2**). Even for the latter LTPs, limited information is currently available on the initial trigger and how this trigger alters kinases and/or phosphatases to appropriately modify LTPs. The atomic mechanisms underlying the phospho-regulation of LTPs have also not yet been elucidated. Therefore, the 3D structures of full-size LTPs consisting of multiple domains and motifs need to be investigated in more detail. To the best of our knowledge, the 3D structures of full-size LTPs composed of multiple domains and disordered regions have not yet been elucidated in detail, whereas various domains isolated from LTPs are now known. A more challenging goal may be to obtain high-resolution 3D structures of MCS or MCS-mimicking complexes, in which an LTP bridges specific partners of different organelles (e.g., a tripartite complex of CERT or OSBP with membrane-embedded VAP and PI4P). Important insights into the regulation of LTPs may also be provided by system biological approaches with genetic and pharmacological tools, which may reveal the landscape of kinase/phosphatase networks in living cells (209, 210).

**TABLE 2.** Phospho-regulation of mammalian LTPs

Human LTP	Phospho-Regulation	References
CERT (GPBPΔ26, STARD11)	Hyper-phosphorylation of the SRM inhibits the activities of the PH and START domains of CERT (see also the text)	(73–75)
	Phosphorylation of Ser315 enhances the affinity of CERT for VAP (see also the text)	(72)
StAR (STARD1)	Phosphorylation of Ser195 (Ser194 in the mouse) by protein kinase A upregulates its steroidogenic function	(227, 228)
	Phosphorylation of Ser232 by ERK1 may modulate StAR	(229)
OSBP	Multisite phosphorylation of OSBP regulates sterol enclosing	(230)
PCTP (STARD2)	Phosphorylation of Ser110 (possibly by protein kinase C) induces its mitochondrial relocation	(231)
PITPβ (PI/SM-TP)	Phosphorylation of Ser262 (possibly by protein kinase C) induces its Golgi relocation	(232)
STARD10	Phosphorylation of Ser284 by casein kinase 2 reduces its affinity to membranes and down-regulates its PC/PE transfer activity	(233)
ORP3	Hyper-phosphorylation of ORP3 enhances its affinity for VAP	(234)

Alternative names of LTPs are indicated in parentheses. PCTP, PC-transfer protein; PITPβ, PITP β isoform; PI/SM-TP, PI/SM-transfer protein.

Cytosolic LTPs are topologically inaccessible to lipids oriented to the luminal side of organelles. Hence, it will be important to elucidate whether and how LTP-mediated inter-membrane transport systems coordinate with intra-membrane transport systems and inter-organelle vesicular transport systems in the network of lipid trafficking events. It also remains to be clarified whether and how multiple LTP-mediated pathways redundantly contribute to inter-organelle transport of a specific lipid type in a cell.

The list of LTP or LTD members has been growing as whole genome sequences in various organisms are analyzed by rapidly advanced bioinformatic tools. However, it has sometimes been difficult to envision lipid transport properties for some of these proteins. For instance, proteins with the lipid-transfer START domain in plants often contain the DNA-binding homeobox domain (211), which suggests their role as transcription factors with yet to be identified lipid ligands as their regulators. Thus, it may also be interesting to revisit the physiological roles of proteins that have been annotated as LTPs. As summarized in previous review articles (63, 93, 212–215), LTPs may have functions beyond inter-organelle lipid transport. For instance, the Sec14 protein, which is required for transport of secretory proteins from the *trans*-Golgi network in yeast, has the activity of inter-membrane transfer of PI (216). It later turned out that the primary role of Sec14 is not inter-membrane transfer of lipids, but rather as a regulator of the yeast Golgi-recruiting PI-4 kinase, Pik1, by changing the conformation of Sec14 depending on bound lipid ligands (214). Some LTPs known as typical “lipid-carriers” appear to have dual functions: for example, cytosolic OSBP forms a complex with protein phosphatases, depending on cholesterol (217). ORP5 interacts with mTORC complex 1, a central regulator of cell proliferation (218).

LTPs are now regarded as new types of medical molecular targets in human health and disease. Nevertheless, chemical inhibitors of LTPs have not been developed, except for a few LTPs that include CERT (219, 220), StAR (221), and OSBP (as well as ORPs) (222–224). Thus, new LTP inhibitors as pharmacological tools are desired not only for research but also for medical applications.

The names of several LTDs reflect their lipid substrates that were initially identified for the respective LTDs. However, the nomenclature of LTDs after specific lipid types sometimes causes confusion when it represents the family of the domains because various members of an LTD recognize different lipid types (as exemplified by PS transfer by “oxysterol”-binding protein-related domains and C1P transfer by the glycolipid-transfer domain). This issue needs to be resolved in the future, although the historical background needs to be appreciated.

In addition, it will also be crucial to elucidate the general mechanistic principle of LTP biology. To achieve this, the aforementioned proposal that LTPs mediate the rectified and accurate inter-organelle fluxes of lipids at MCSs indirectly coupling with metabolic reactions may provide several testable questions: for example, *i*) Does inhibition of the anabolic conversion of a “forward-transported” lipid in its targeting organelle suppress the backward transport of its coexchangeable lipid (and vice versa)? *ii*) Does the elimination of organelle-binding modules (such as the PH domain and FFAT motif in CERT and OSBP) result in a loss of accuracy in delivery (and then an increase in misdelivery to other organelles)? *iii*) Does the shortening or removal of “disordered” regions between organelle-binding modules impair the function of the LTP for the inter-organelle transport of lipids without any detrimental effects on the inter-membrane transfer activity of its LTD? A previous study elucidated some aspects of the predicted disordered region between an ER transmembrane helix and a VASt domain (141). The intellectual and material resources that have accumulated over the past decade will facilitate further study of intracellular lipid trafficking events in the decade to come.
